# Supplementary material for: Prenylated Flavonoids with Potential Antimicrobial Activity: Synthesis, Biological Activity, and In Silico Study
Source: Int J Mol Sci. 2021 May 22;22(11):5472. doi: 10.3390/ijms22115472 (PMC8196815; doi:10.3390/ijms22115472)
Supplement: Supplementary file 1 [file ijms-22-05472-s001.zip › ijms-1218291-supplementary.pdf]

Synthesis and Antimicrobial Activity of Prenylated Flavonoids

Mauricio Osorio[1*], Marcela Carvajal[2], Alejandra Vergara[2], Estefania Butassi[3], Susana Zacchino[3], Carolina Mascayano[4], Margarita Montoya[5], Sophia Mejías[5], Marcelo Cortez-San Martín[6], and Yesseny Vásquez-Martínez[7*]

^{1}H- and ^{13}C –NMR spectra of obtained prenylated compounds

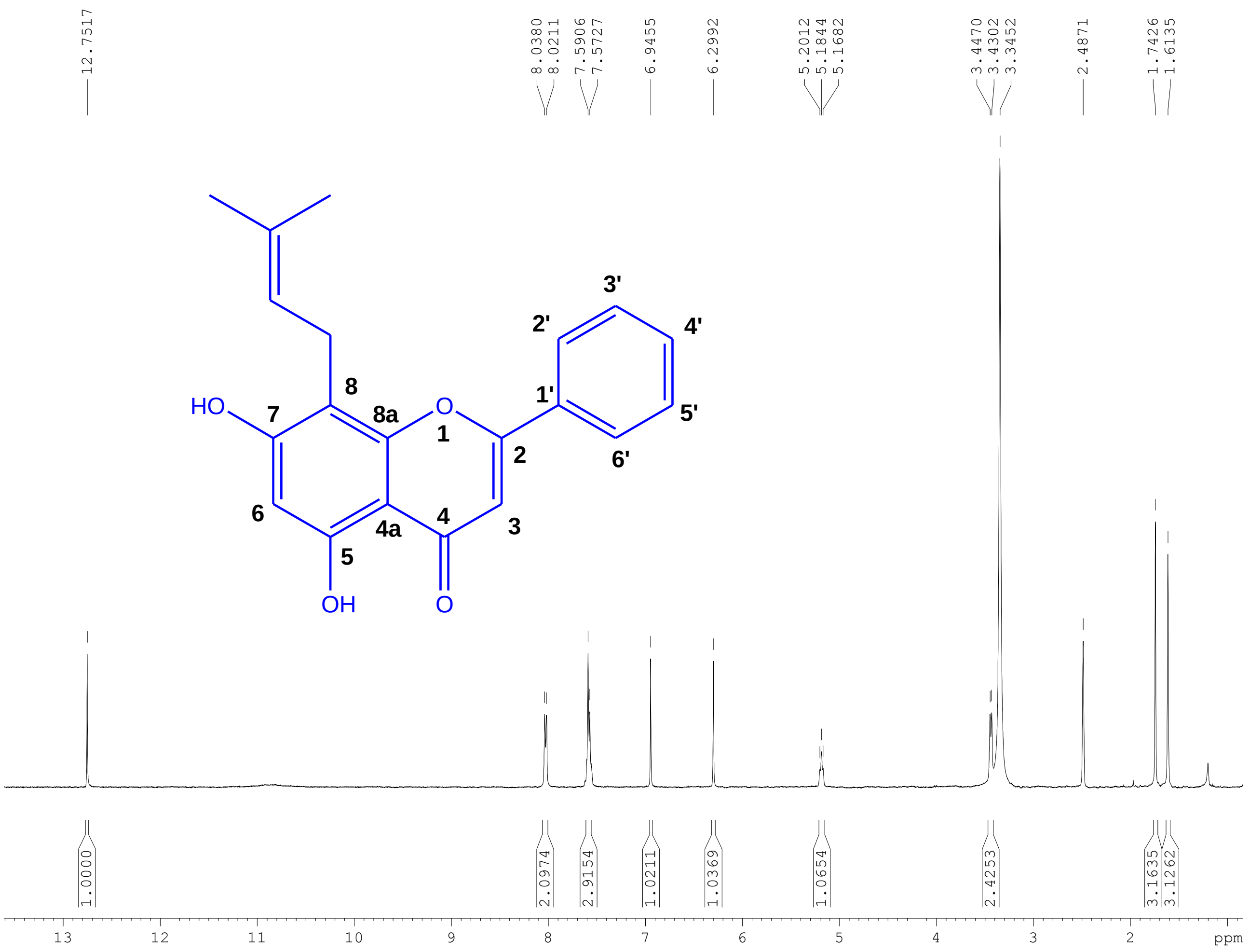

Figure S1. ^{1}H NMR (400 MHz, DMSO-d_6) spectrum of **7**. δ ppm: 12.75 (s, 1H, ArO*H*-5); 10.6 – 11.0 (br s, 1H, ArO*H*-7); 8.03 (d, 2H, J = 6.8 Hz, 2′,6′-Ar*H*); 7.59-7.57 (m, 3H, 3′,4′,5′-Ar*H*); 6.95 (s, 1H, Ar*H*-3); 6.30 (s, 1H, Ar*H*-6); 5.18 (br t, 1H, C*H*=C(CH$_3$)$_2$); 3.44 (d, 2H, J = 6.7 Hz, C*H*$_2$CH=C(CH$_3$)$_2$); 1.74 (s, 3H, -CH$_2$CH=CC*H*$_3$CH$_3$); 1.61 (s, 3H, -CH$_2$CH=CCH$_3$C*H*$_3$).

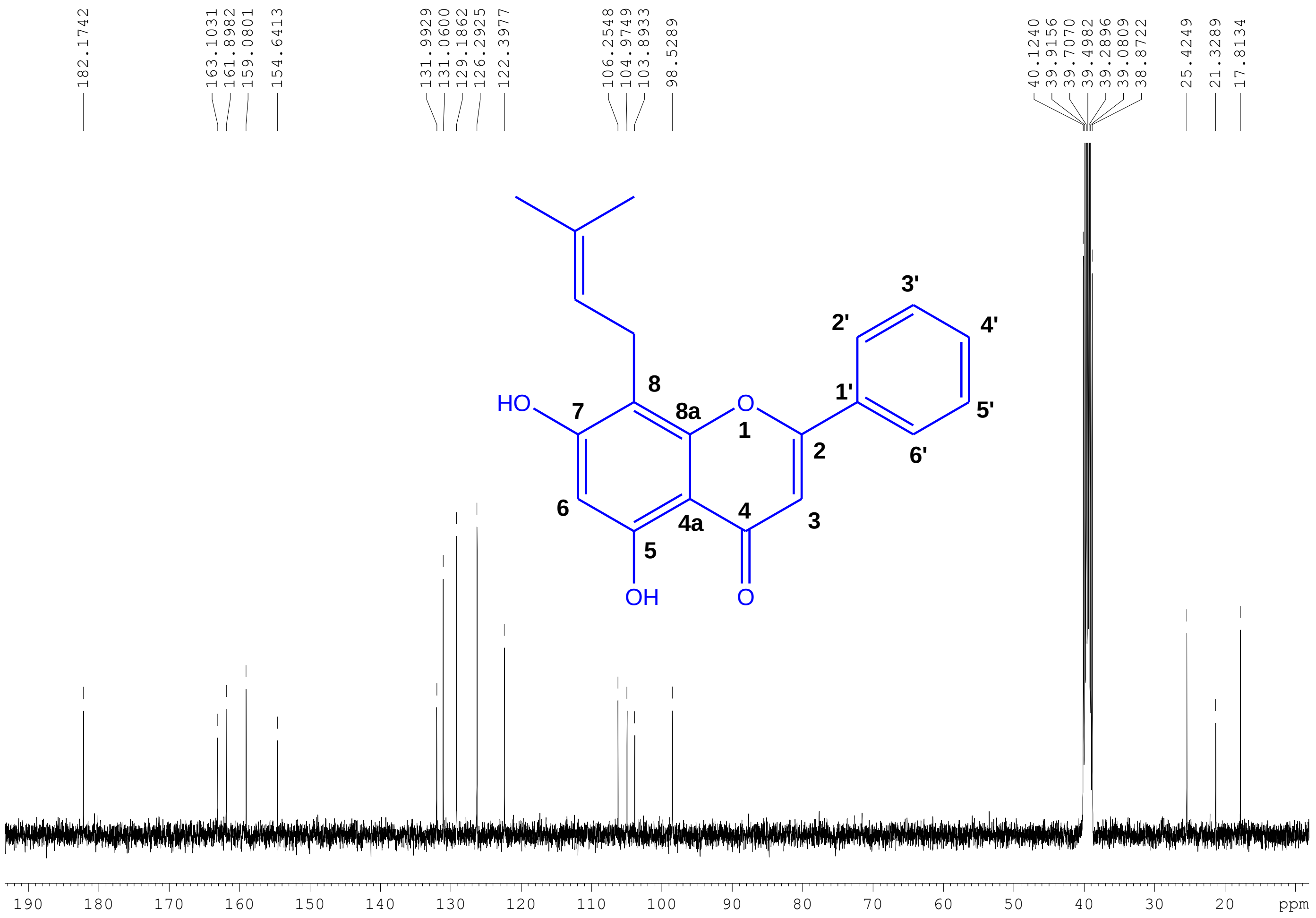

Figure S2. ^{13}C NMR (100 MHz, DMSO-d_6) spectrum of **7**. δ ppm:17.8 (-CH=CCH_3CH_3); 21.3 (-CH_2CH=CCH_3CH_3); 25.4 (CH_2CH=CCH_3CH_3); 98.5 (Ar*C*-6); 103.9 (Ar*C*-4a); 105.0 (Ar*C*-3); 106.3 (Ar*C*-8); 122.4 (*C*H=C($CH_3)_2$); 126.3 (Ar*C*-2′,6′); 129.2 (Ar*C*-3′,5′); 131.1 (CH=*C*($CH_3)_2$); 132.0 (Ar*C*-4′); 154.6 (Ar*C*-8a); 159.1 (Ar*C*-5); 161.9 (Ar*C*-7); 163.1 (Ar*C*-2); 182.2 (4-*C*=O).

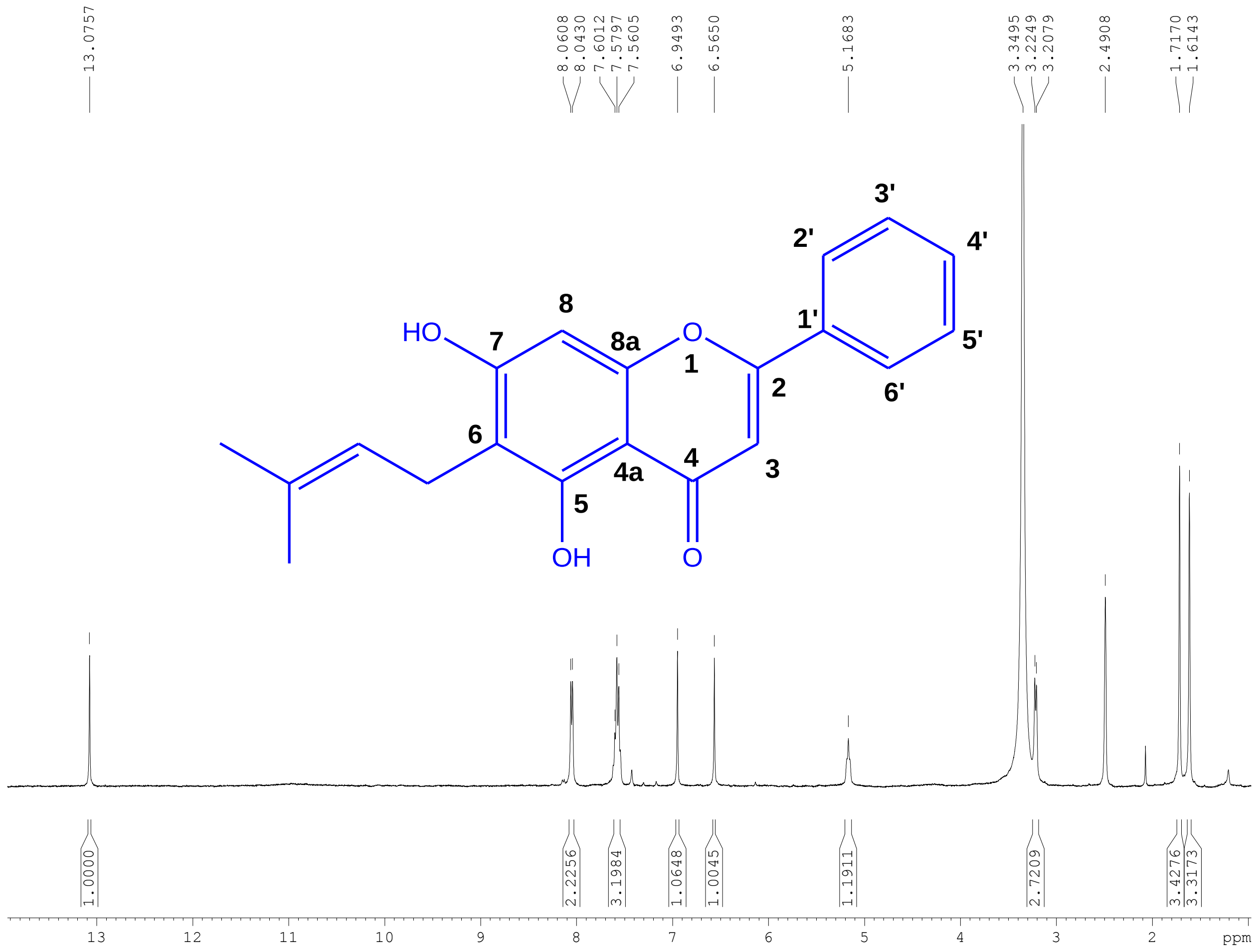

Figure S3. ^{1}H NMR (400 MHz, DMSO-d_6) spectrum of **8**. δ ppm: 13.08 (s, 1H, ArO*H*-5); 8.05 (d, 2H, J = 7.1 Hz, 2′,6′-Ar*H*); 7.60-7.56 (m, 3H, 3′,4′,5′-Ar*H*); 6.95 (s, 1H, Ar*H*-3); 6.57 (s, 1H, Ar*H*-6); 5.17 (br t, 1H, C*H*=C(CH_3)$_2$); 3.22 (d, 2H, J = 6.8 Hz, C*H*$_2$CH=C(CH_3)$_2$); 1.72 (s, 3H, -CH_2CH=CC*H*$_3$$CH_3$); 1.61 (s, 3H, -$CH_2$CH=C$CH_3$C*H*$_3$).

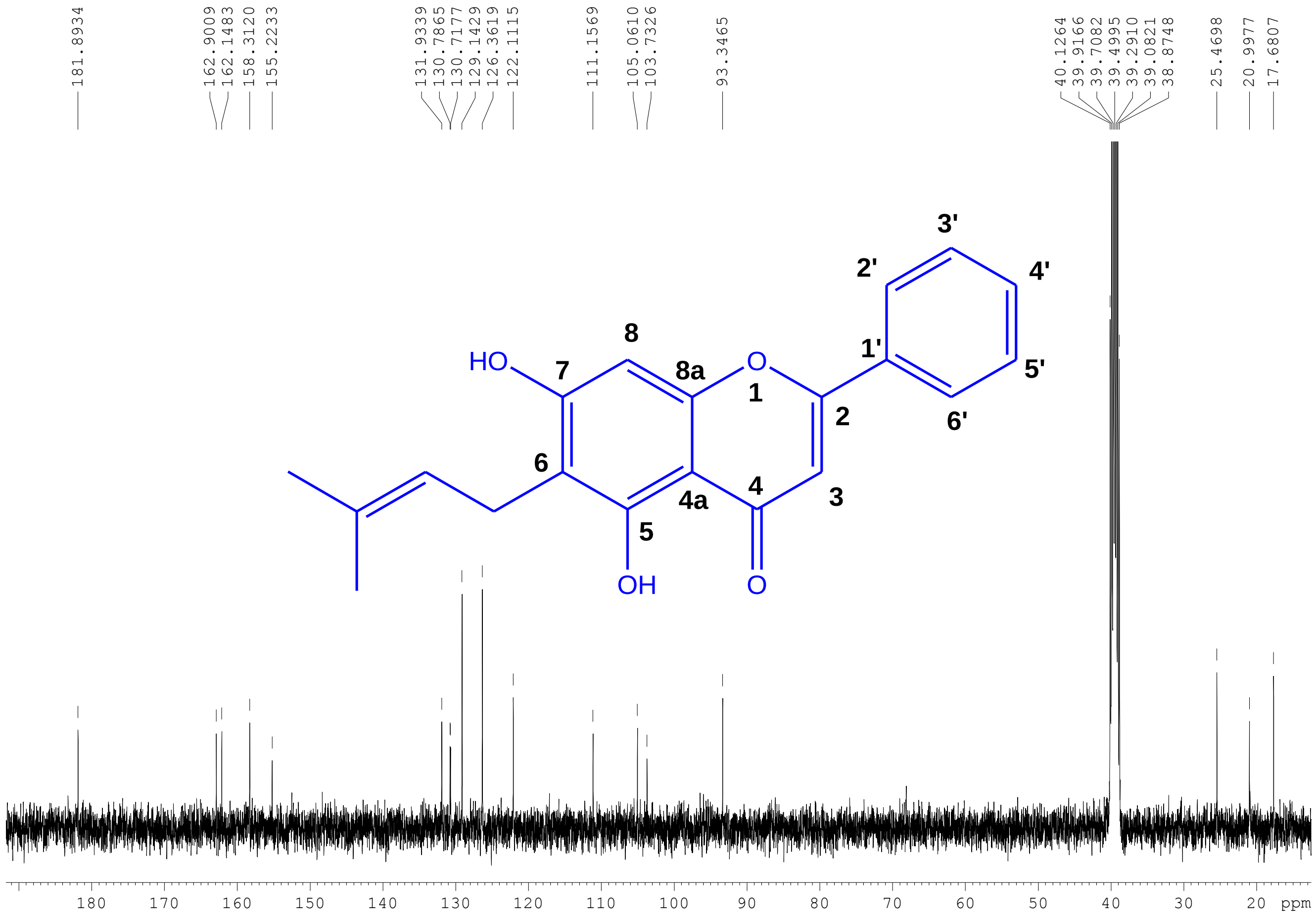

Figure S4. ^{13}C NMR (100 MHz, DMSO-*d*$_6$) spectrum of **8**. δ ppm:17.7 (-CH=C*C*H$_3$CH$_3$); 21.0 (-*C*H$_2$CH=CCH$_3$CH$_3$); 25.5 (CH$_2$CH=CCH$_3$*C*H$_3$); 93.4 (Ar*C*-8); 103.7 (Ar*C*-4a); 105.0 (Ar*C*-3); 111.2 (Ar*C*-6); 122.1 (*C*H=C(CH$_3$)$_2$); 126.4 (Ar*C*-2′,6′); 129.1 (Ar*C*-3′,5′); 130.7 (CH=*C*(CH$_3$)$_2$); 130.8 (Ar*C*-1′); 131.9 (Ar*C*-4′); 155.2 (Ar*C*-8a); 158.3 (Ar*C*-5); 162.2 (Ar*C*-7); 162.9 (Ar*C*-2); 181.9 (4-*C*=O).

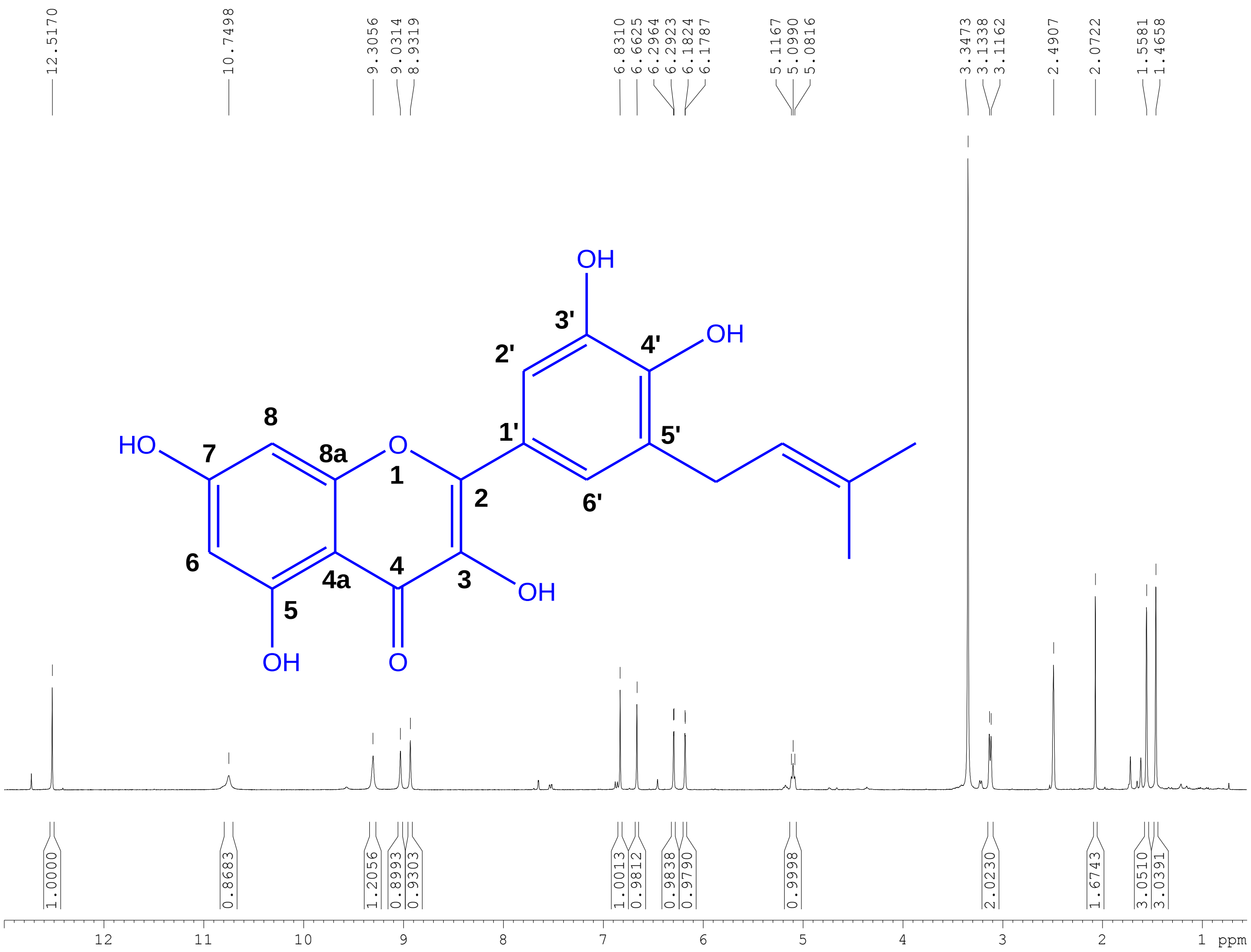

Figure S5. ^{1}H NMR (400 MHz, DMSO-d_6) spectrum of **9**. δ ppm: 12.52 (s, 1H, ArO*H*-5); 10.75 (br s, 1H, ArO*H*-7); 9.31 (s, 1H, ArO*H*-3'); 9.03 (s, 1H, ArO*H*-4'); 8.93 (s, 1H, ArO*H*-3); 6.83 (s, 1H, Ar*H*-2'); 6.66 (s, 1H, Ar*H*-6'); 6.29 (d, 1H, J=1.6 Hz, Ar*H*-8); 6.18 (d, 1H, J=1.5.Hz, Ar*H*-6); 5.10 (br t, 1H, C*H*=C(CH$_3$)$_2$); 3.12 (d, 2H, J=7.0 Hz, C*H*$_2$CH=C(CH$_3$)$_2$); 1.56 (s, 3H, CH$_2$CH=CC*H*$_3$CH$_3$); 1.47 (s, 3H, CH$_2$CH=CCH$_3$C*H*$_3$).

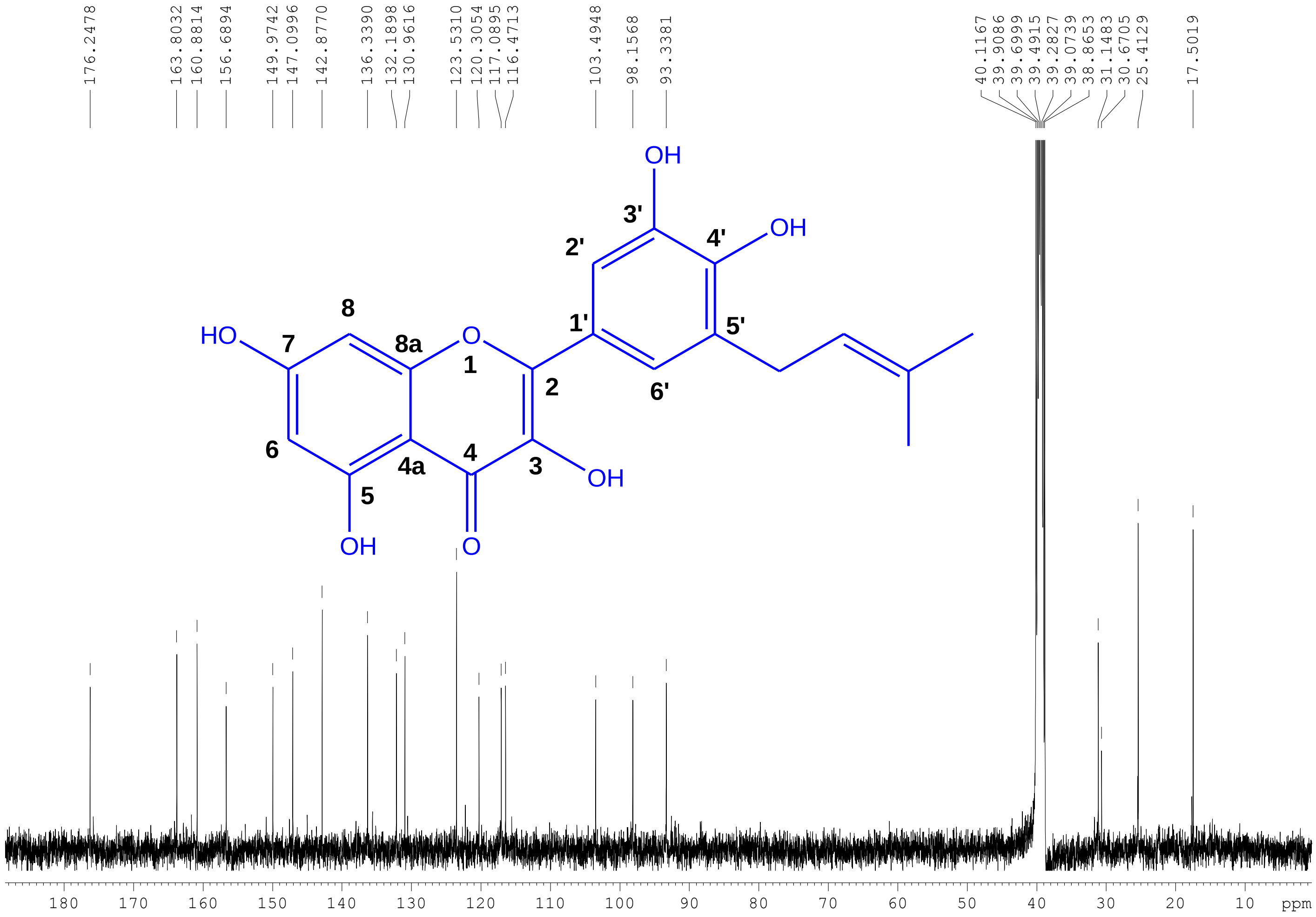

Figure S6. ^{13}C NMR (100 MHz, DMSO-d_6) spectrum of **9**. δ ppm:17.5 (CH=CCH$_3$CH$_3$); 25.4 (CH$_2$CH=CCH$_3$CH$_3$); 31.1 (*C*H$_2$CH=CCH$_3$CH$_3$); 93.3 (Ar*C*-8); 98.2 (Ar*C*-6); 103.5 (Ar*C*-4a); 116.5 (Ar*C*-6′); 117.1 (Ar*C*-2′); 120.3 (Ar*C*-5′); 123.5 (*C*H=C(CH$_3$)$_2$); 131.0 (CH=*C*(CH$_3$)$_2$); 132.2 (Ar*C*-3′); 136.3 (Ar*C*-2); 142.9 (Ar*C*-1′); 147.1 (Ar*C*-4′); 150.0 (Ar*C*-3); 156.7 (Ar*C*-8a); 160.9 (Ar*C*-5); 163.8 (Ar*C*-7); 176.2 (4-*C*=O).

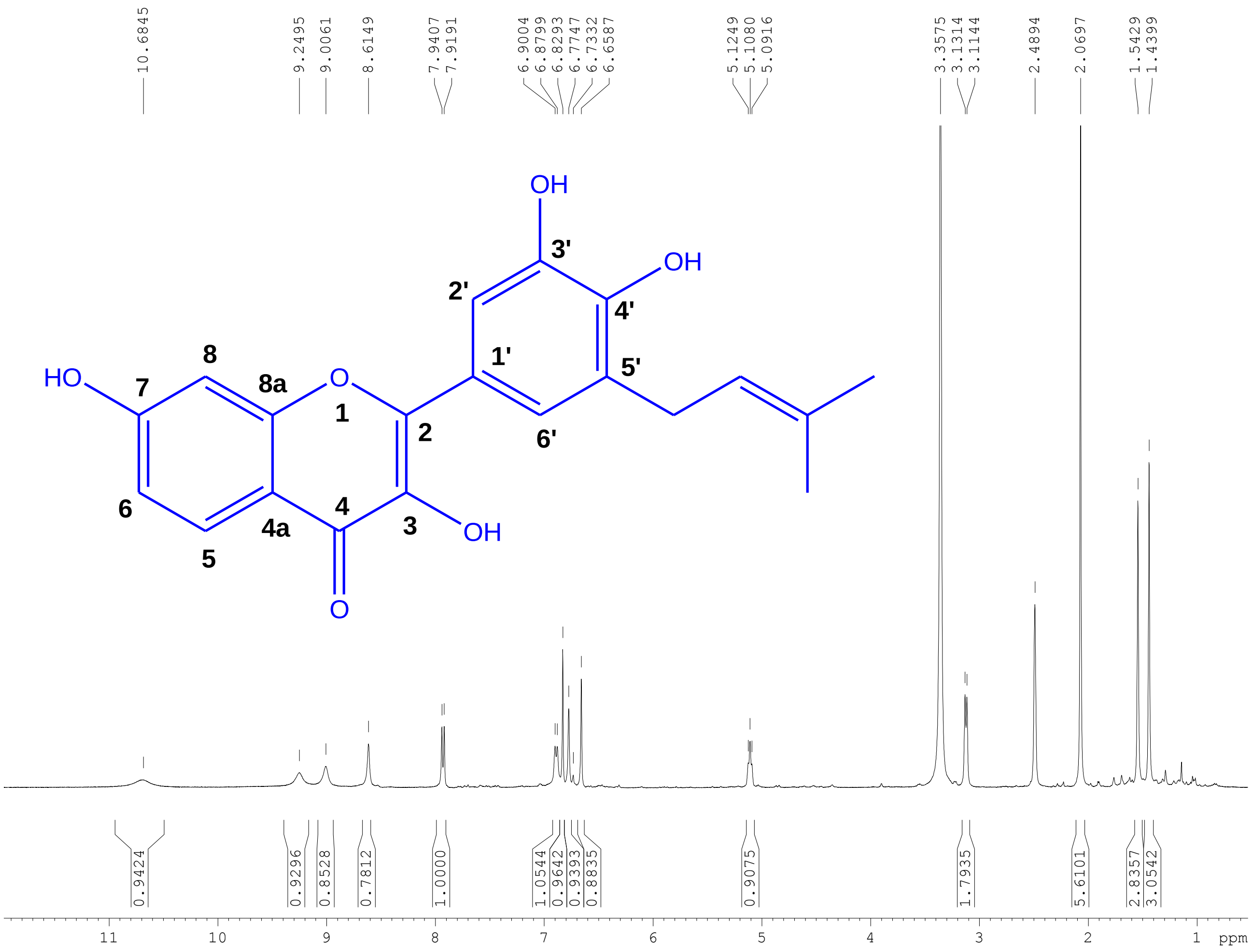

Figure S7. ^{1}H NMR (400 MHz, DMSO-d_6) spectrum of **10**. δ ppm: 10.68 (br s, 1H, ArO*H*-7); 9.25 (s, 1H, ArO*H*-3'); 9.01 (s, 1H, ArO*H*-4'); 8.62 (s, 1H, ArO*H*-3); 7.93 (d, 1H, J = 8.6 Hz, Ar*H*-5); 6.89 (d, 1H, J = 8.2 Hz, Ar*H*-6); 6.83 (s, 1H, Ar*H*-2'); 6.78 (s, 1H, Ar*H*-8); 6.66 (s, 1H, Ar*H*-6'); 5.11 (br t, 1H, C*H*=C(CH_3)$_2$); 3.12 (d, 2H, J=6.8 Hz, C*H*$_2$CH=C(CH_3)$_2$); 1.54 (s, 3H, CH_2CH=CC*H*$_3$$CH_3$); 1.44 (s, 3H, CH_2CH=CCH_3C*H*$_3$).

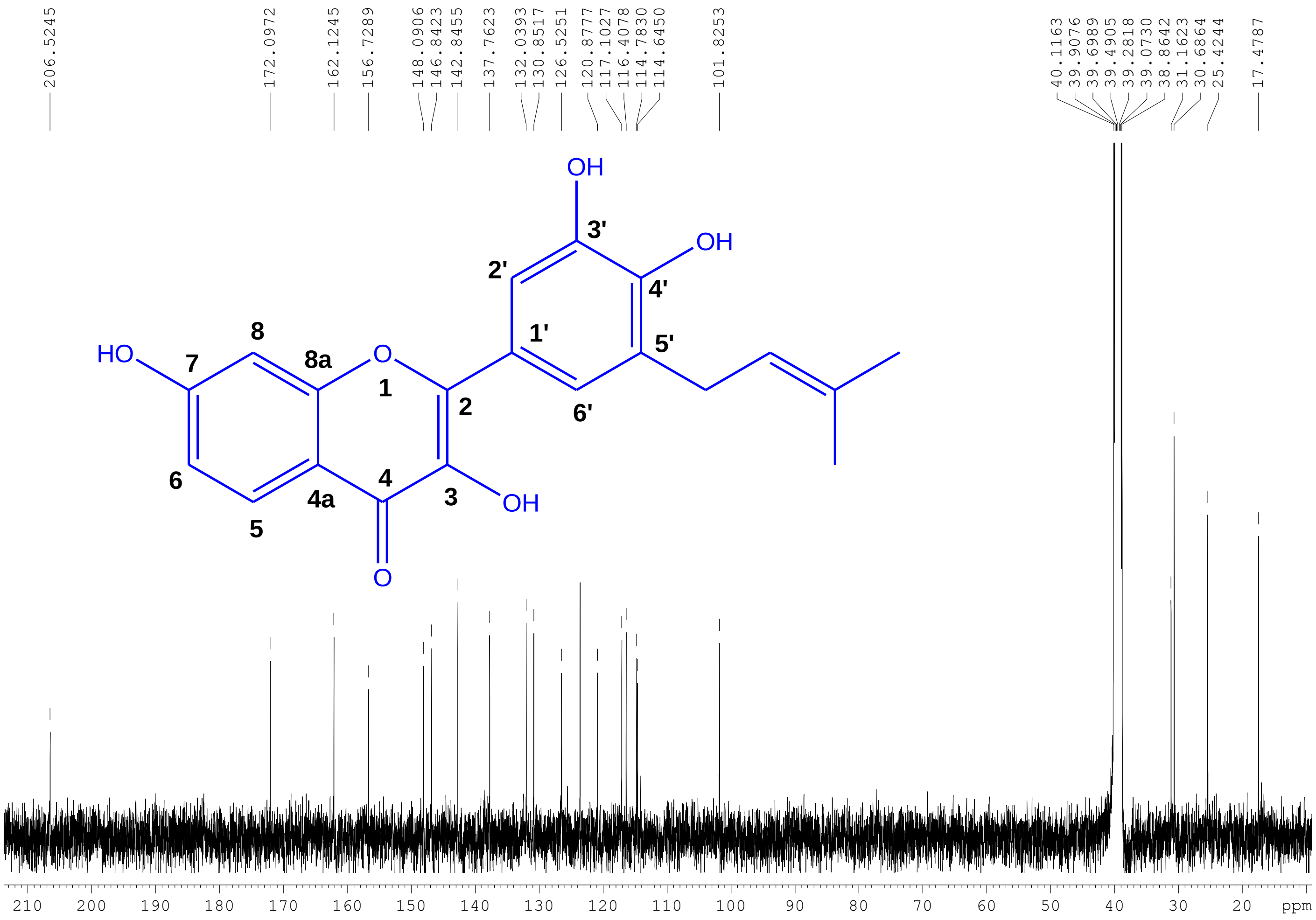

Figure S8. ^{13}C NMR (100 MHz, DMSO-*d*$_6$) spectrum of **10.** δ ppm: 17.5 (CH=CCH_3CH_3); 25.4 (CH_2CH=CCH_3CH_3); 31.2 (CH_2CH=CCH_3CH_3); 101.8 (Ar*C*-8);114.6 (Ar*C*-6); 114.8 (Ar*C*-4a); 116.4 (Ar*C*-6′); 117.1 (Ar*C*-2′); 120.9 (Ar*C*-5′); 123.6 (*C*H=C(CH_3)$_2$); 126.5 (Ar*C*-5); 130.8 (CH=*C*(CH_3)$_2$); 132.0 (Ar*C*-3′); 137.8 (Ar*C*-1′); 142.8 (Ar*C*-2); 146.8 (Ar*C*-4′); 148.1 (Ar*C*-3); 156.7 (Ar*C*-8a); 162.1 (Ar*C*-7); 172.1 (4-*C*=O).

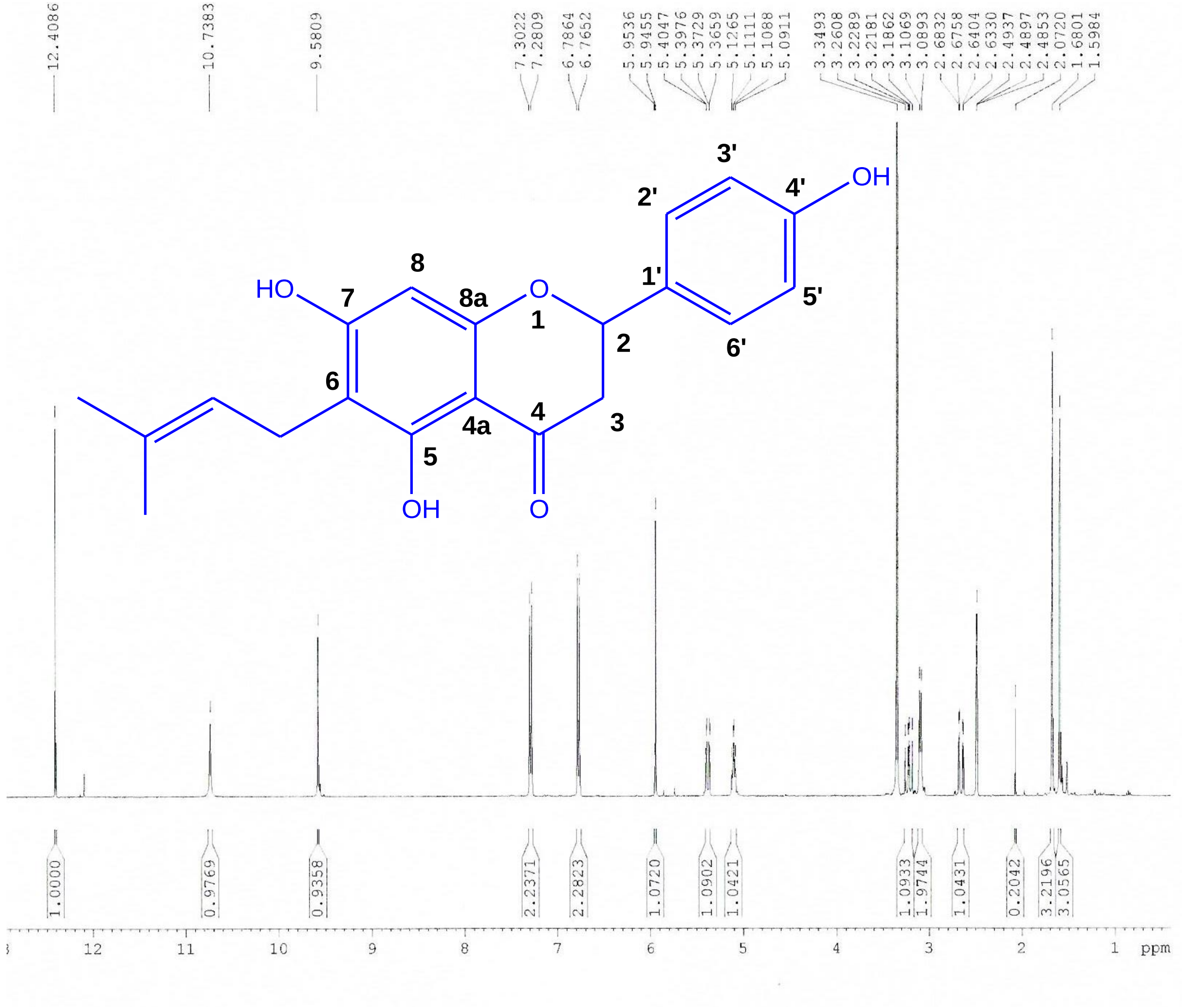

Figure S9. ^{1}H NMR (400 MHz, DMSO-d_6) spectrum of **11**. δ ppm: 12.40 (s, 1H, Ar-O*H*-5); 10.74 (br s, 1H, ArO*H*-7); 9.55 (br s, 1H, ArO*H*-4′); 7.29 (d, 2H, J = 8.4 Hz, Ar*H*-2′,6′); 6.78 (d, J = 8.4 Hz, 2H, Ar*H*-3′,5′); 5.94 (s, 1H, Ar*H*-8); 5.39 (dd, 1H, J_1 = 12.5 Hz, J_2 = 2.6 Hz, C*H*-2); 5.11 (br t, 1H, C*H*=C(CH_3)$_2$); 3.22 (dd, 1H, J_1 = 17.1 Hz, J_2 = 12.7 Hz, CH*H*-3); 3.10 (d, 2H, J = 7.0 Hz, C*H*$_2$CH=C(CH_3)$_2$); 2.66 (dd, 1H, J_1=14.3 Hz, J_2 = 2.9 Hz, C*H*H-3); 1.68 (s, 3H, CH_2CH=CC*H*$_3$$CH_3$); 1.60 (s, 3H, CH_2CH=CCH_3C*H*$_3$).

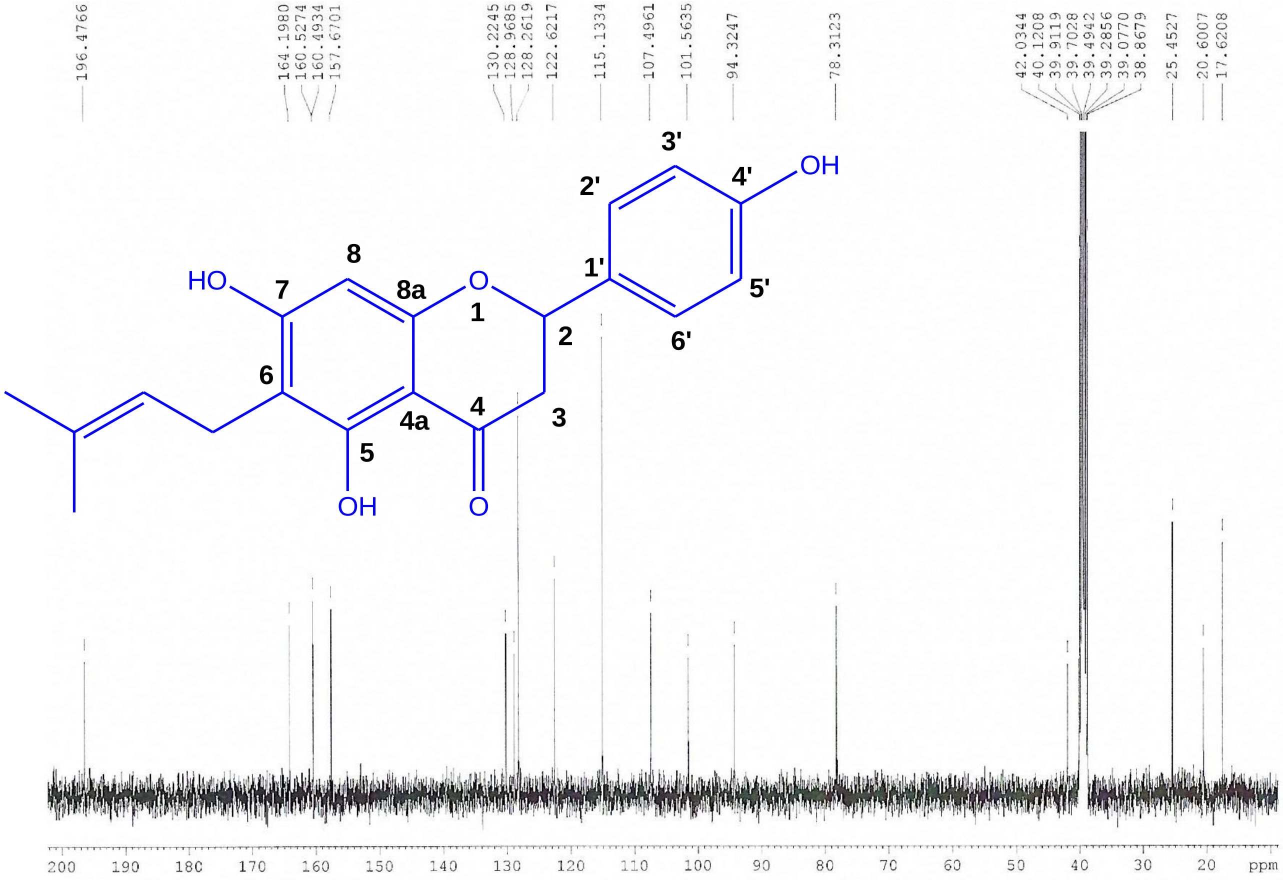

Figure S10. ^{13}C NMR (100 MHz, DMSO-d_6) spectrum of **11**. δ ppm: 17.6 (-CH=CCH$_3$CH$_3$); 20.6 (-CH$_2$CH=CCH$_3$CH$_3$); 25.4 (-CH$_2$CH=CCH$_3$CH$_3$); 42.0 (*C*H$_2$-3); 78.3 (Ar*C*-2); 94.3 (Ar*C*-8); 101.5 (Ar*C*-4a); 107.5 (Ar*C*-6); 115.1 (Ar*C*-3′,5′); 122.6 (*C*H=C(CH$_3$)$_2$); 128.3 (Ar*C*-2′,6′); 128.98 (Ar*C*-1′); 130.2 (CH=*C*(CH$_3$)$_2$); 157.7 (Ar*C*-4′); 160.5 (Ar*C*-7,8a); 164.3 (Ar*C*-5); 196.4 (4-*C*=O).

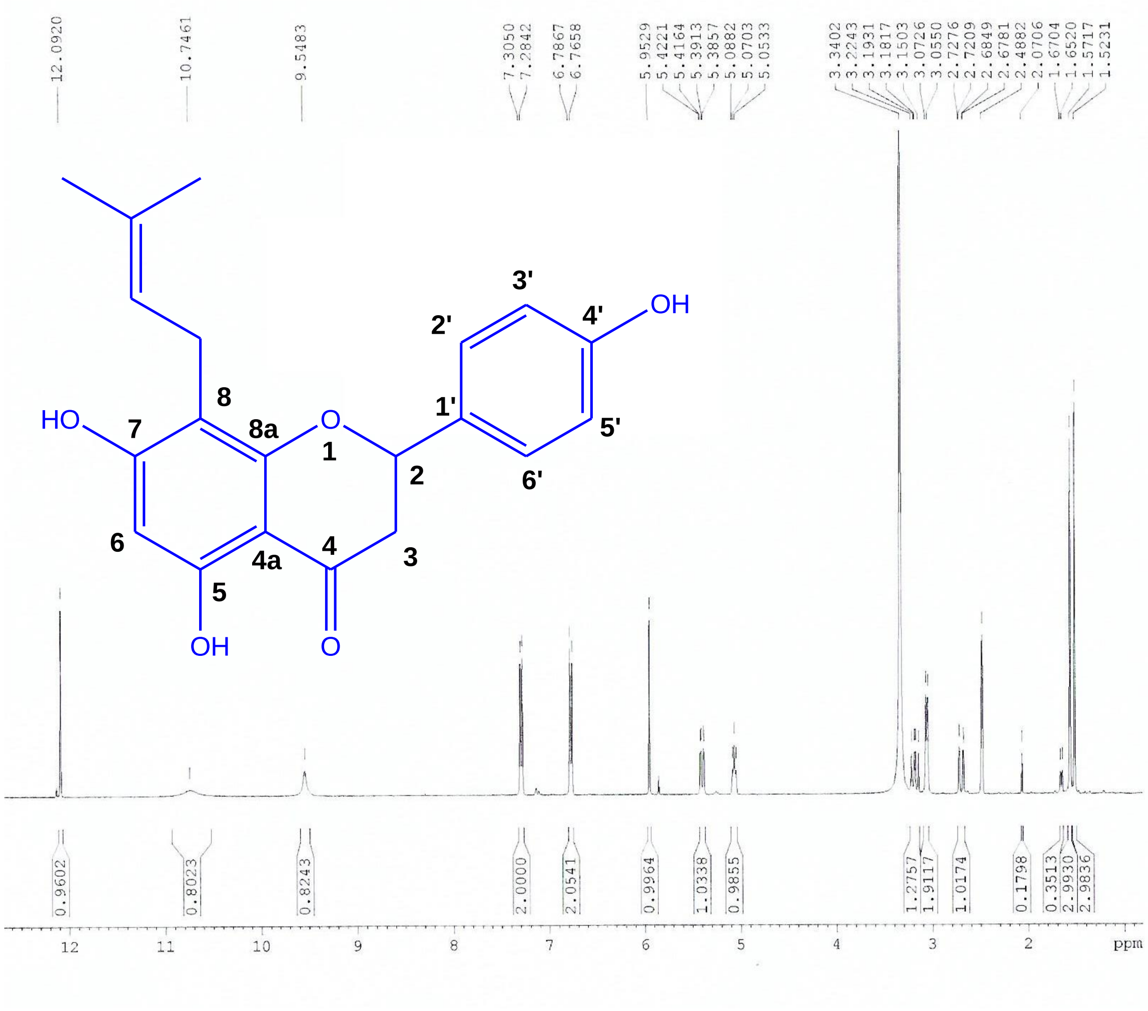

Figure S11. ^{1}H NMR (100 MHz, DMSO-d_6) spectrum of **12**. δ ppm: 12.09 (s, 1H, Ar-O*H*-5); 10.75 (s, 1H, ArO*H*-7); 9.56 (s, 1H, ArO*H*-4') 7.30 (d, 2H, *J* = 8.4 Hz, Ar*H*-2',6'); 6.78 (d, *J* = 8.4 Hz, 2H, Ar*H*-3',5'); 5.95 (s, 1H, Ar*H*-6); 5.40 (dd, 1H, J_1 = 12.4 Hz, J_2 = 2.7 Hz, C*H*-2); 5.07 (br. t, 1H, C*H*=C(CH_3)$_2$); 3.19 (dd, 1H, J_1 = 17.1 Hz, J_2 = 12.6 Hz, CH*H*-3); 3.06 (d, 2H, *J* = 7.1 Hz, CH_2CH=C(CH_3)$_2$); 2.70 (dd, 1H, J_1 = 17.2 Hz, J_2 = 2.7 Hz, C*H*H-3); 1.57 (s, 3H, CH_2CH=CC$H_3$$CH_3$); 1.52 (s, 3H, CH_2CH=CCH_3CH_3).

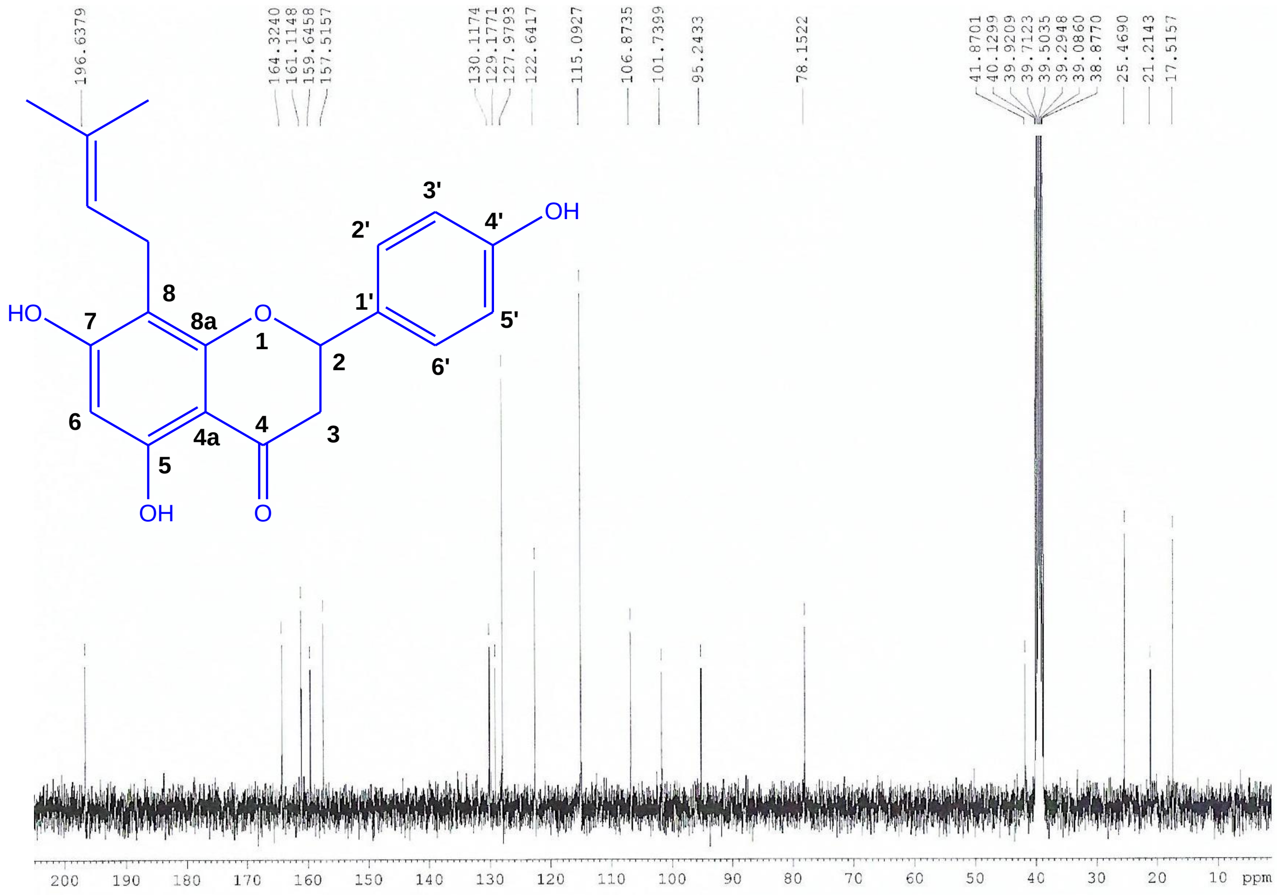

Figure S12. ^{13}C NMR (100 MHz, DMSO-d_6) spectrum of **12**. δ ppm: 17.6 (-CH=CCH$_3$CH$_3$); 21.2 (-CH$_2$CH=CCH$_3$CH$_3$); 25.5 (-CH$_2$CH=CCH$_3$CH$_3$); 41.9 (CH$_2$-3); 78.2 (ArC-2); 95.3 (ArC-6); 101.7 (ArC-4a); 106.9 (ArC-8); 115.1 (ArC-3′,5′); 122.6 (CH=C(CH$_3$)$_2$); 128.0 (ArC-2′,6′); 129.2 (ArC-1′); 130.1 (CH=C(CH$_3$)$_2$); 157.5 (ArC-4′); 159.7 (ArC-8a); 161.1 (ArC-5); 164.3 (ArC-7); 196.6 (4-C=O).

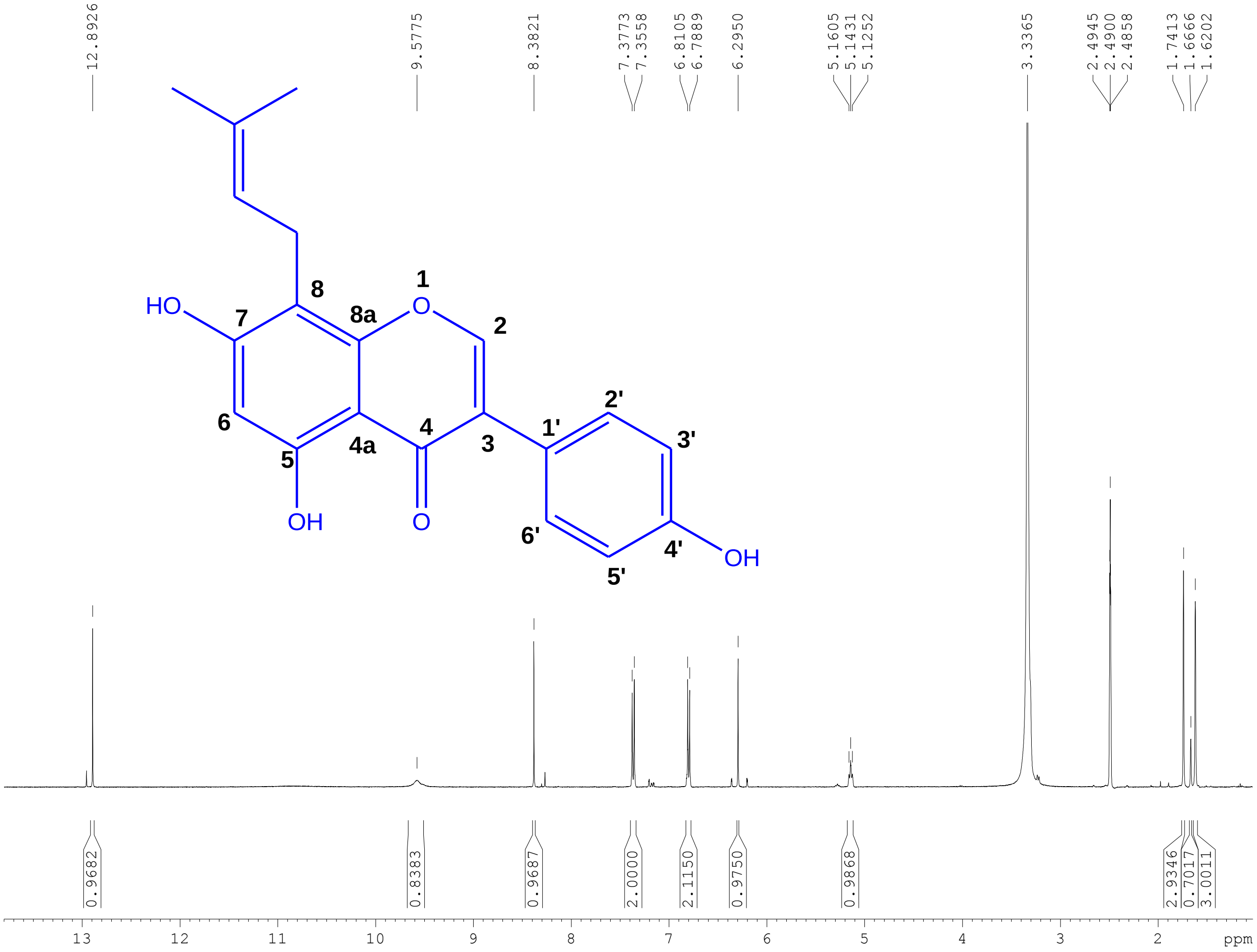

Figure S13. ^{1}H NMR (400 MHz, DMSO-*d*6) spectrum of **13**. δ ppm: 12.98 (s, 1H, ArO*H*-5); 9.58 (br s, 1H, ArO*H*-4′); 8.38 (s, 1H, Ar*H*-2); 7.37 (d, 2H, J = 8.6 Hz, Ar*H*-2′,6′); 6.80 (d, 2H, J = 8.6 Hz, Ar*H*-3′,5′); 6.30 (s, 1H, Ar*H*-6); 5.14 (br t, 1H, C*H*=C(CH_3)$_2$); 3.34 (overload with H_2O, CH_2CH=C(CH_3)$_2$); 1.74 (s, 3H, -CH_2CH=CC$H_3$$CH_3$); 1.62 (s, 3H, -$CH_2$CH=C$CH_3CH_3$).

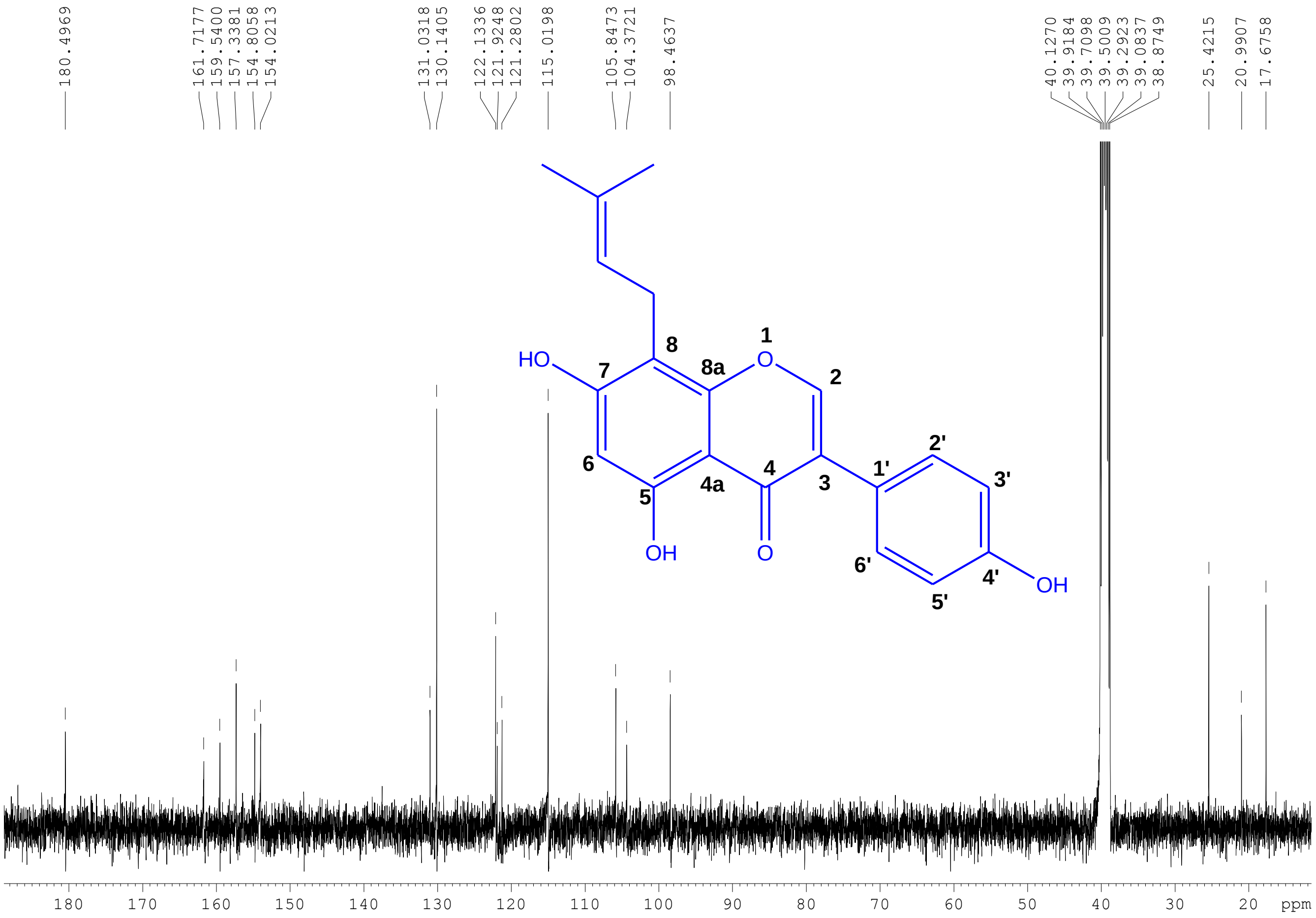

Figure S14. ^{13}C NMR (100 MHz, DMSO-d_6) spectrum of **13**. δ ppm:17.7 (-CH=CCH$_3$CH$_3$); 21.0 (-*C*H$_2$CH=CCH$_3$CH$_3$); 25.4 (CH$_2$CH=CCH$_3$*C*H$_3$); 98.5 (Ar*C*-6); 104.4 (Ar*C*-4a); 105.8 (Ar*C*-8); 115.0 (Ar*C*-3′,5′); 121.3 (Ar*C*-1′); 121.9 (Ar*C*-3); 122.1 (*C*H=C(CH$_3$)$_2$); 130.1 (Ar*C*-2′,6′); 131.0 (CH=*C*(CH$_3$)$_2$); 154.0 (Ar*C*-2); 154.8 (Ar*C*-8a); 157.3 (Ar*C*-4′); 159.5 (Ar*C*-5); 161.7 (Ar*C*-7); 180.5 (4-*C*=O).

2D NMR spectra

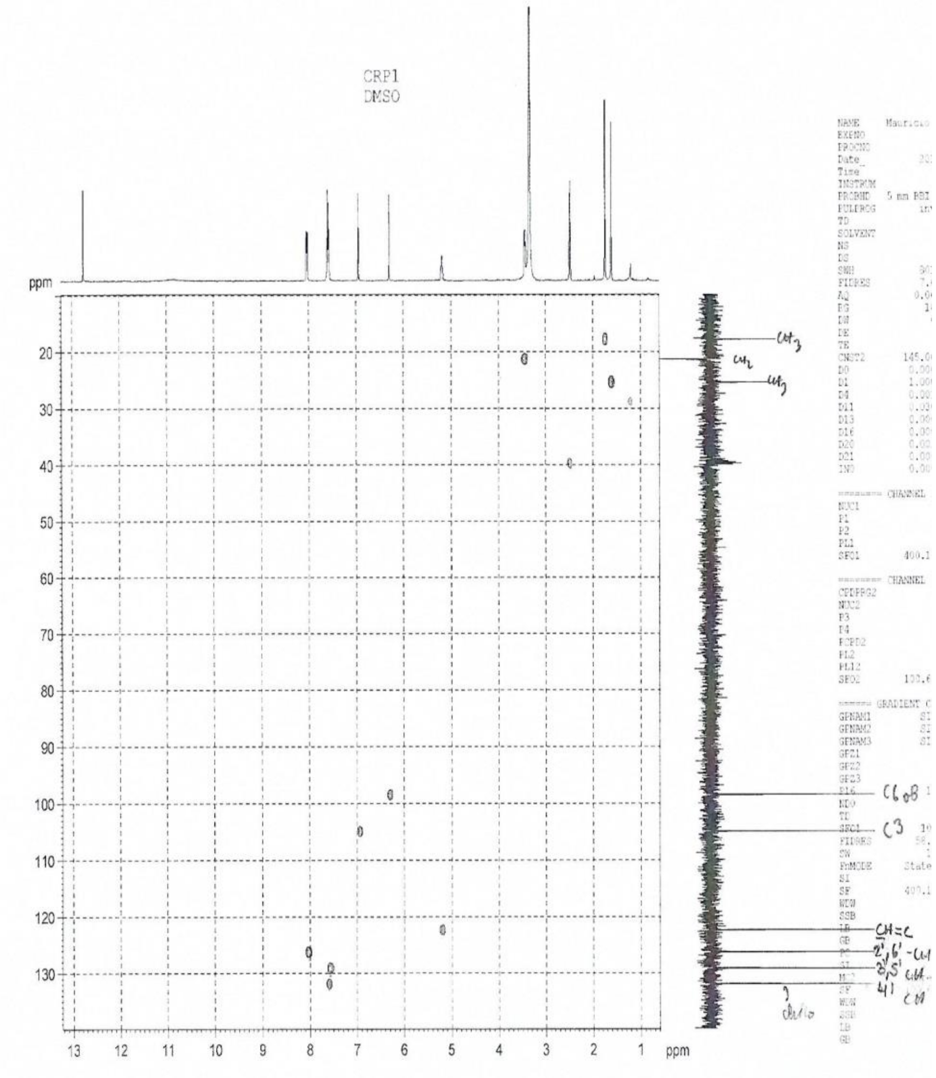

Figure S15. 2D HSQC of **7**.

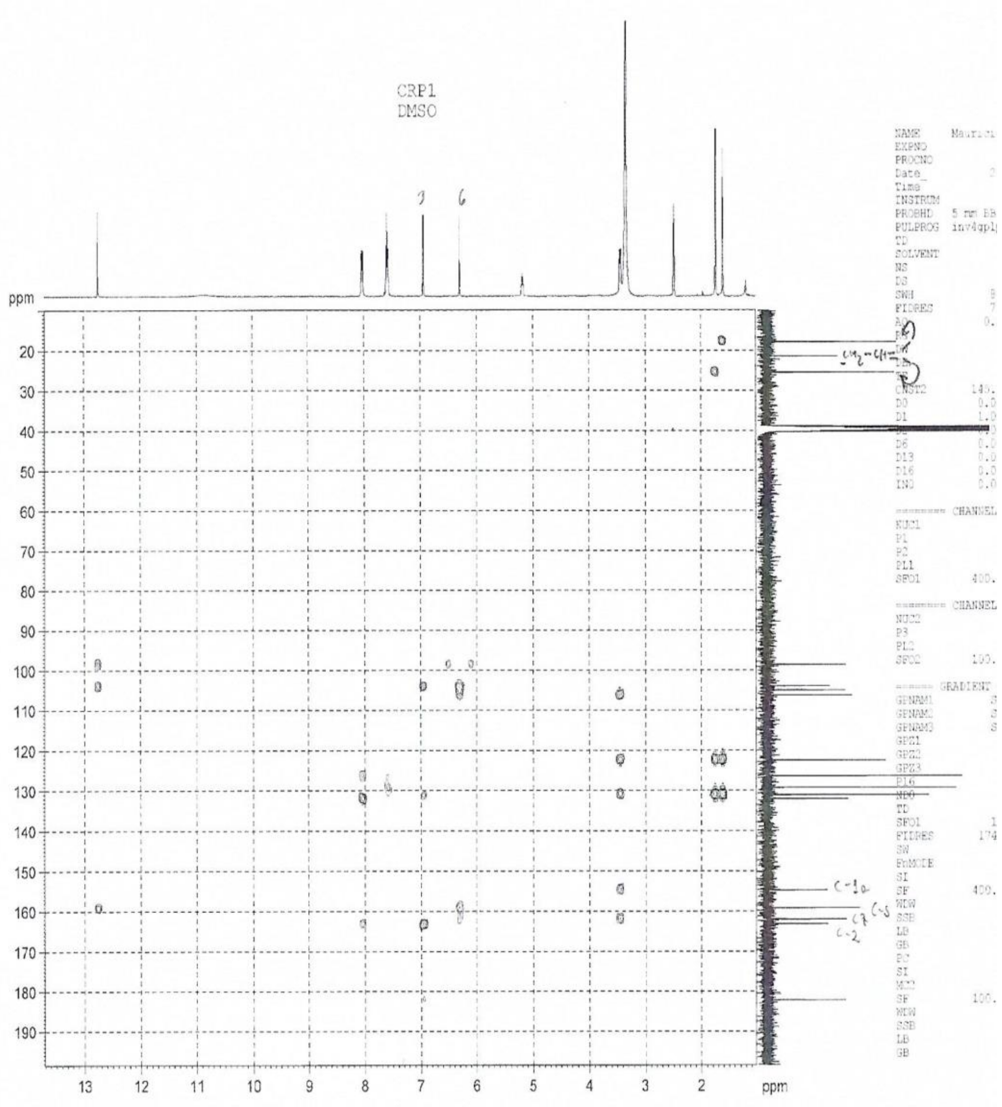

Figure S16. 2D HMBC of **7**.

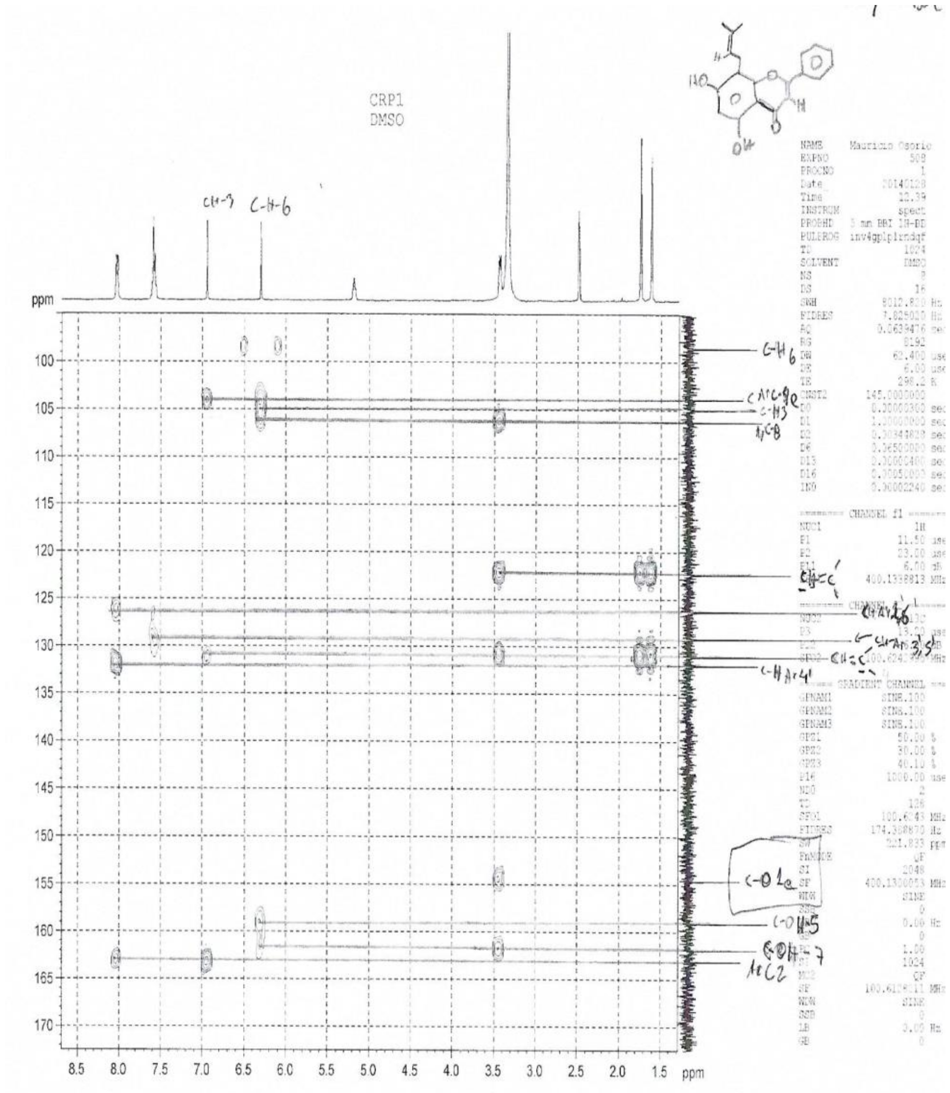

Figure S17. Zoom of 2D HMBC of **7**.

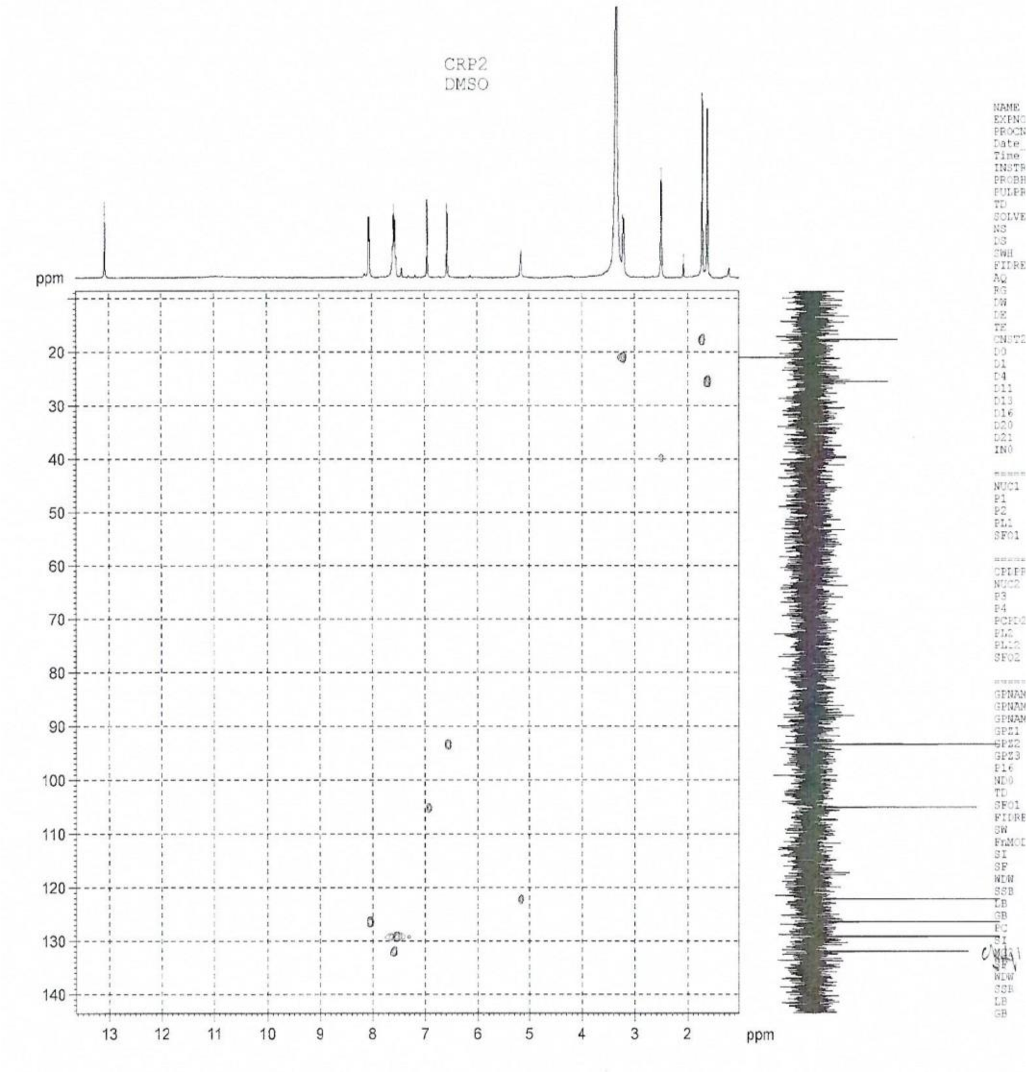

Figure S18. 2D HSQC of **8**.

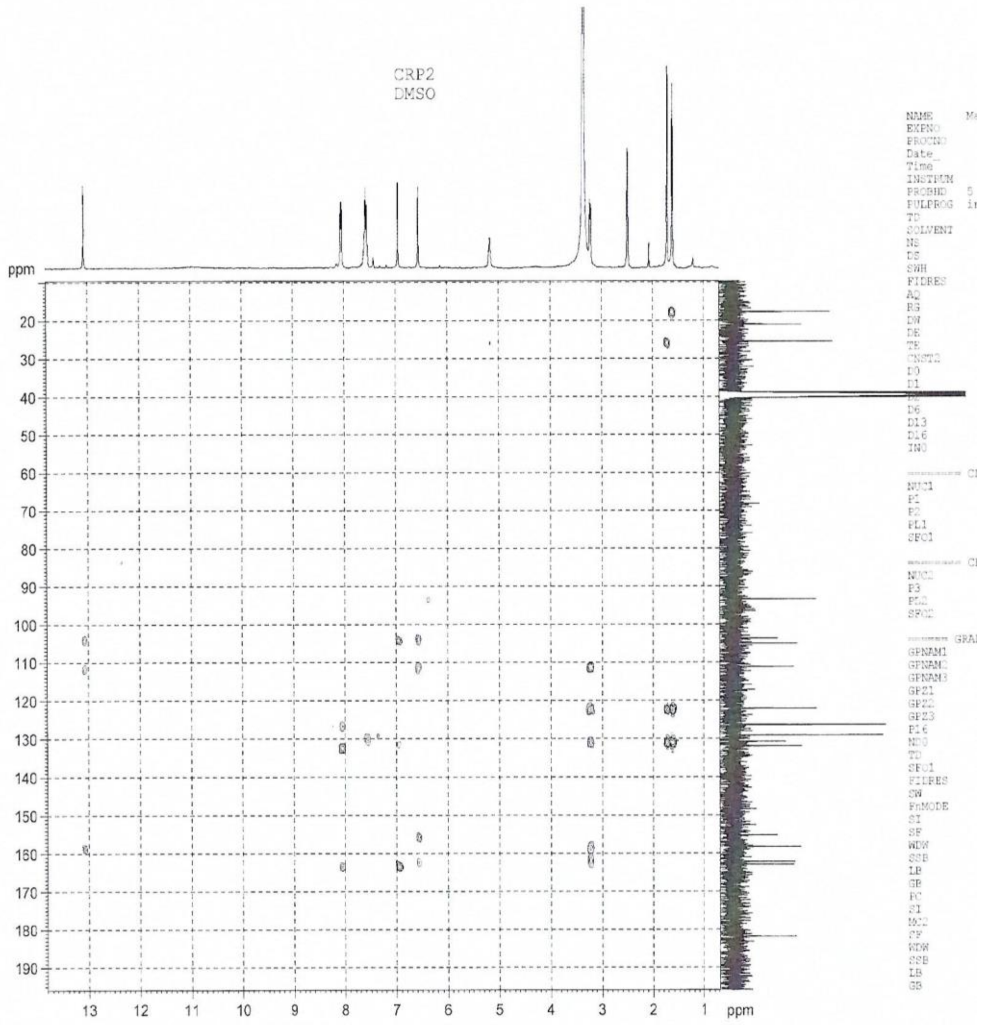

Figure S19. 2D HMBC of **8**.

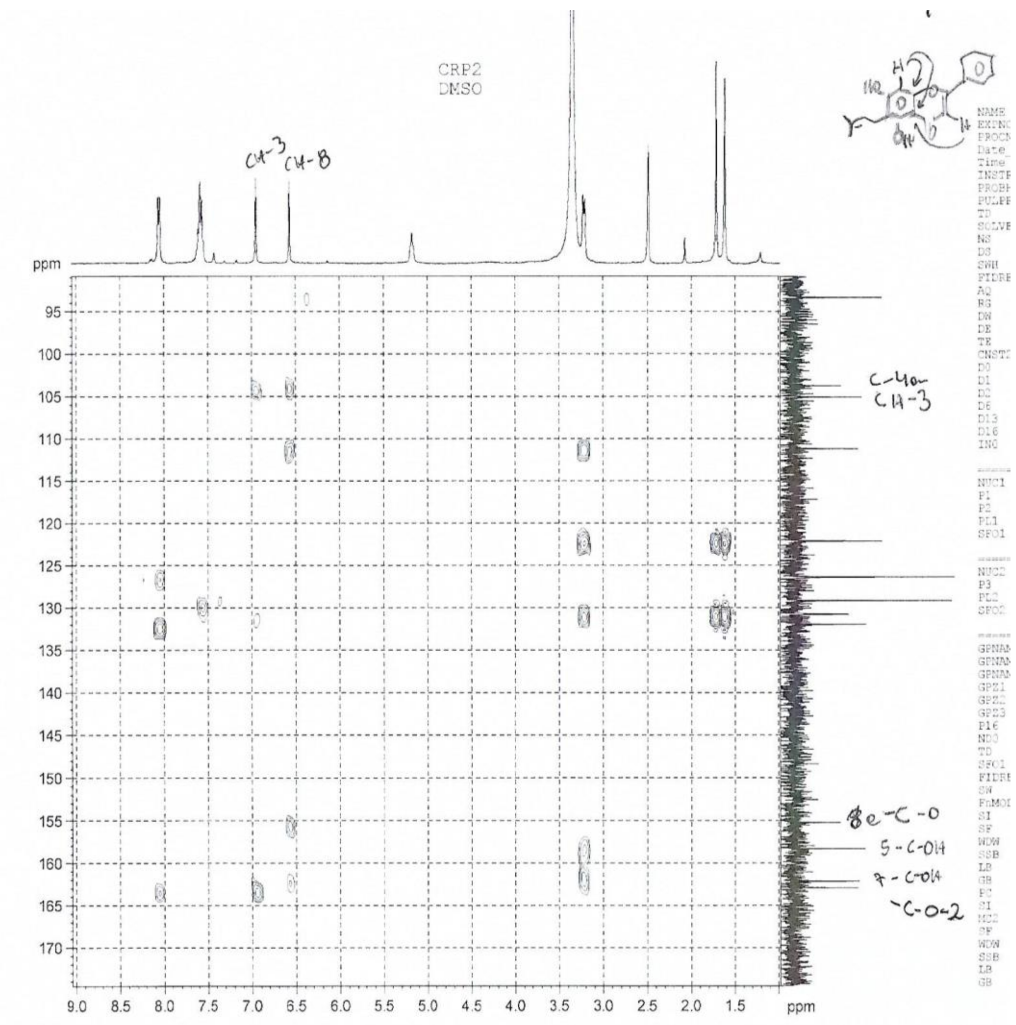

Figure S20. Zoom of 2D HMBC of **8**.

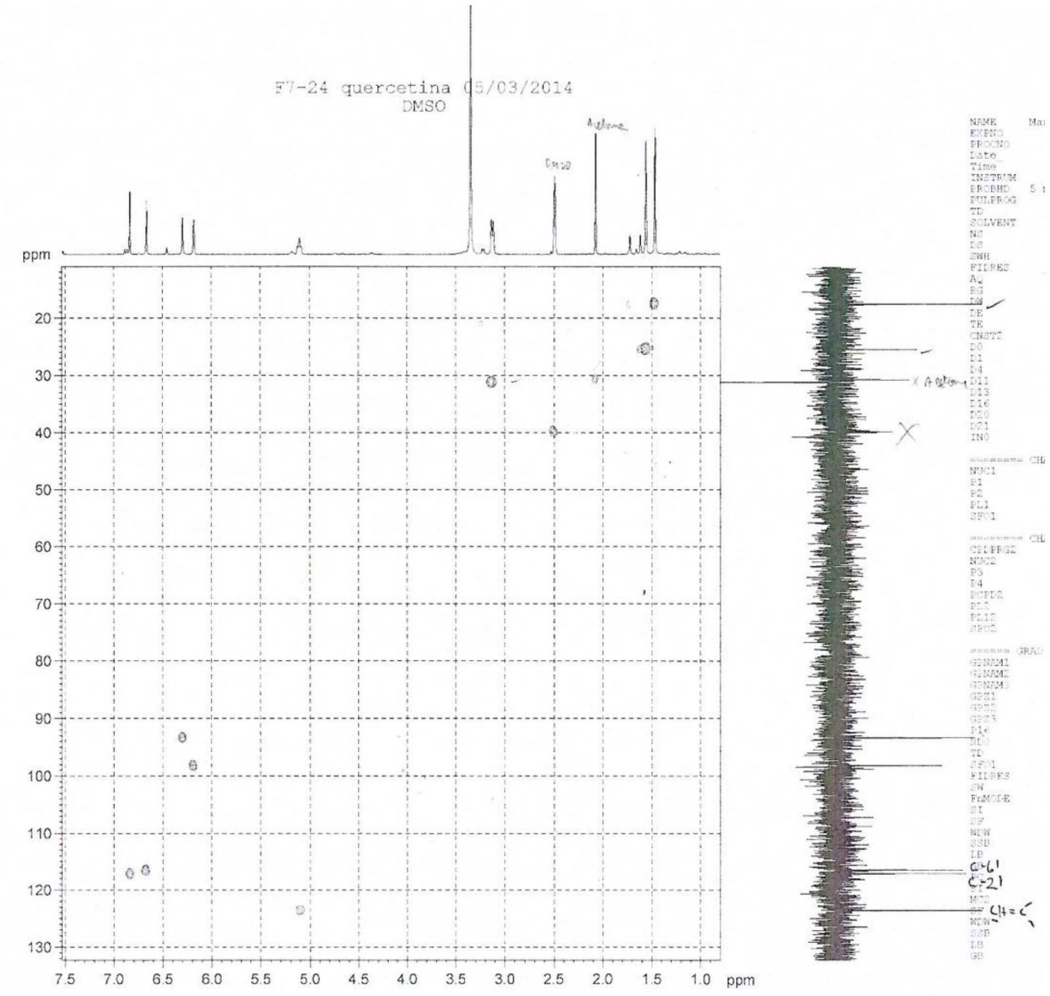

Figure S21. 2D HSQC of **9**.

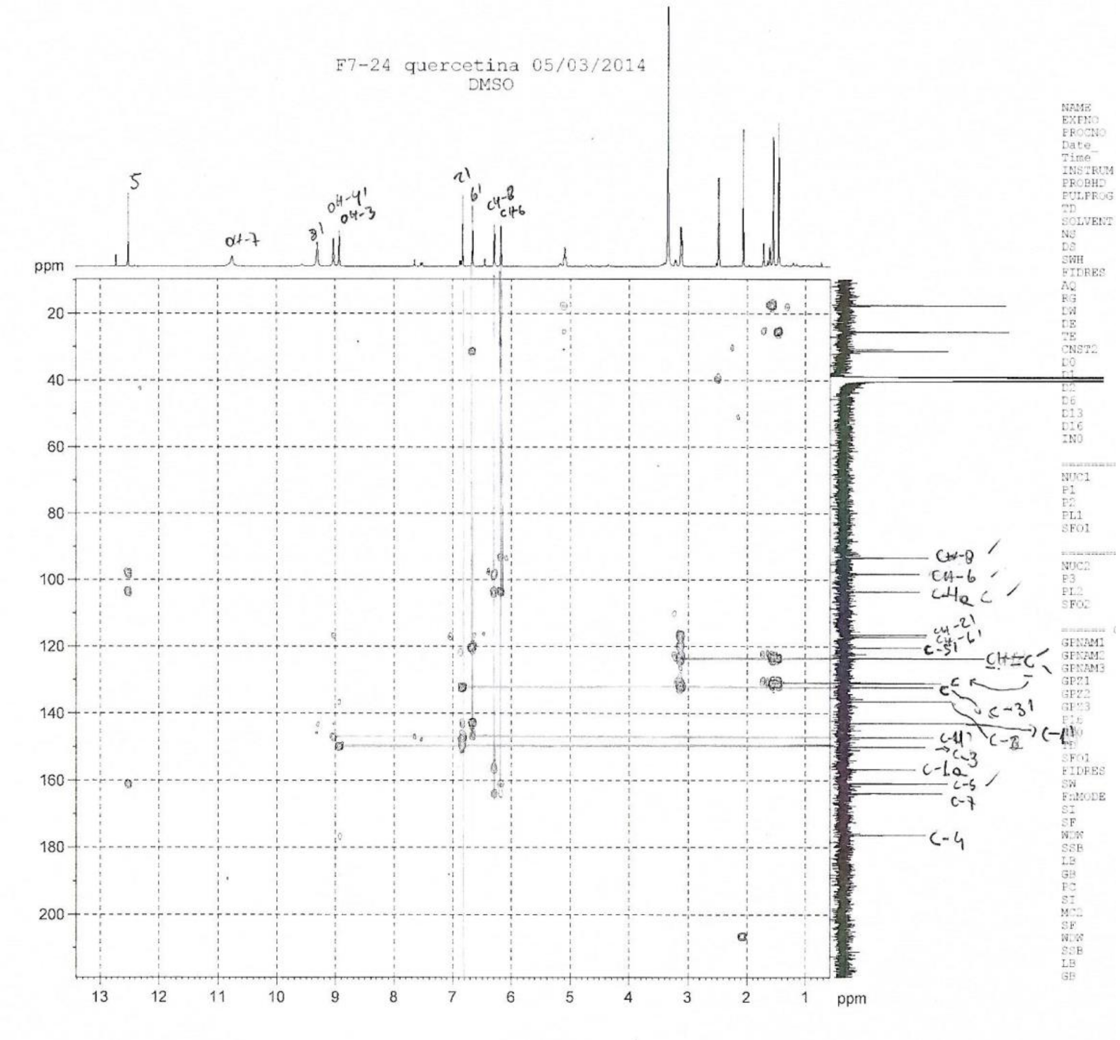

Figure S22. 2D HMBC of **9**.

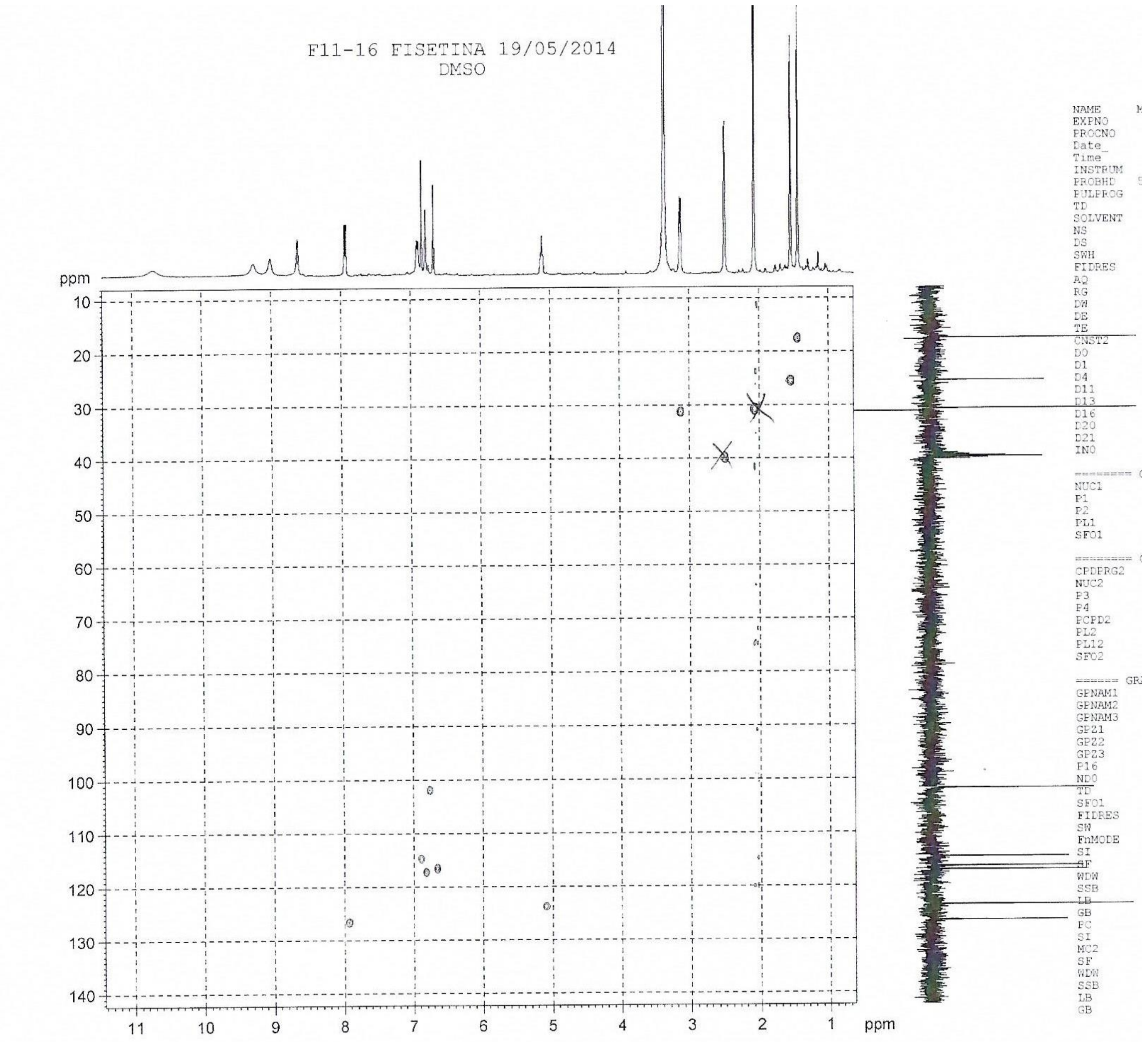

Figure S23. 2D HSQC of **10**.

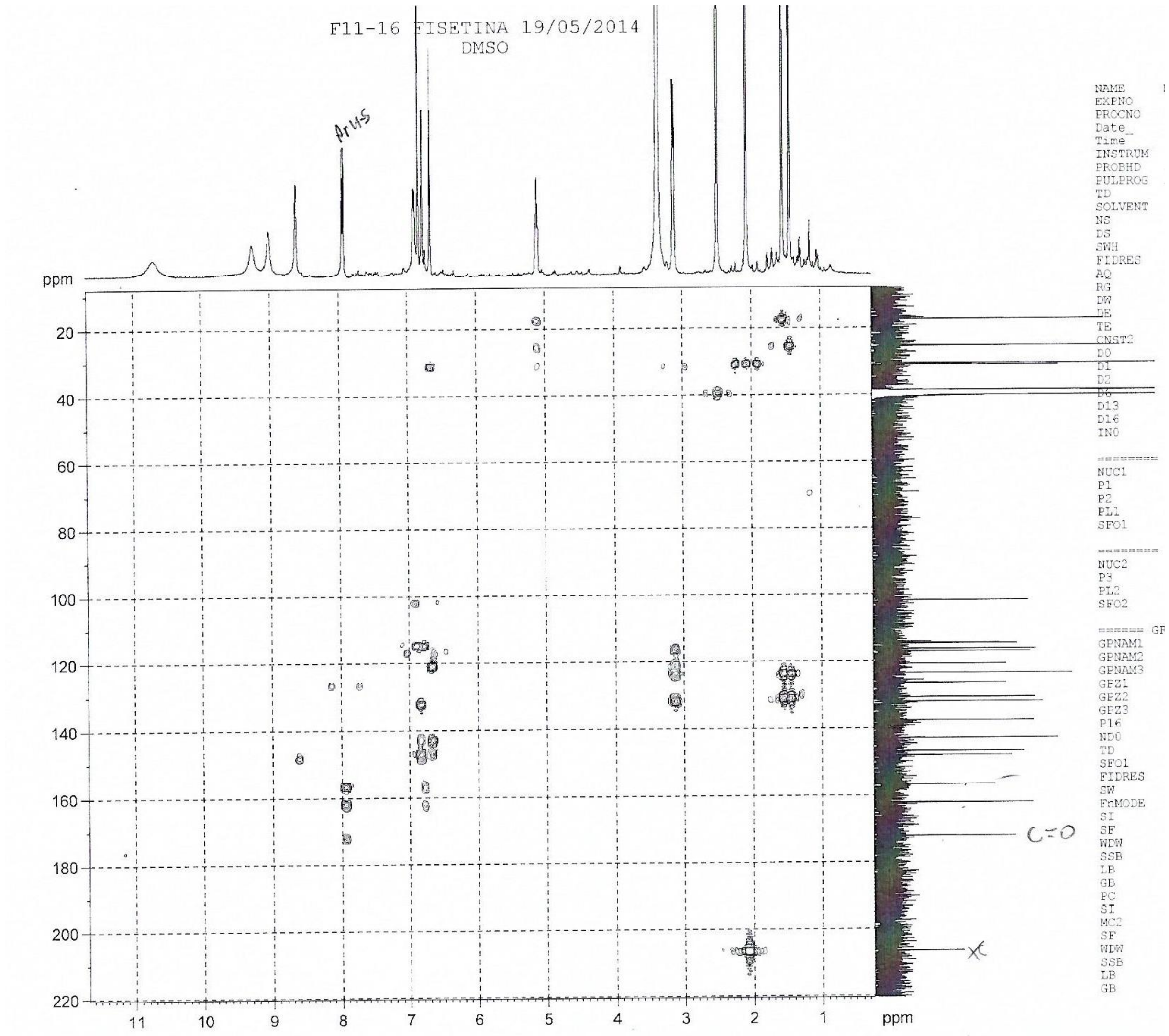

Figure S24. 2D HMBC of **10**.

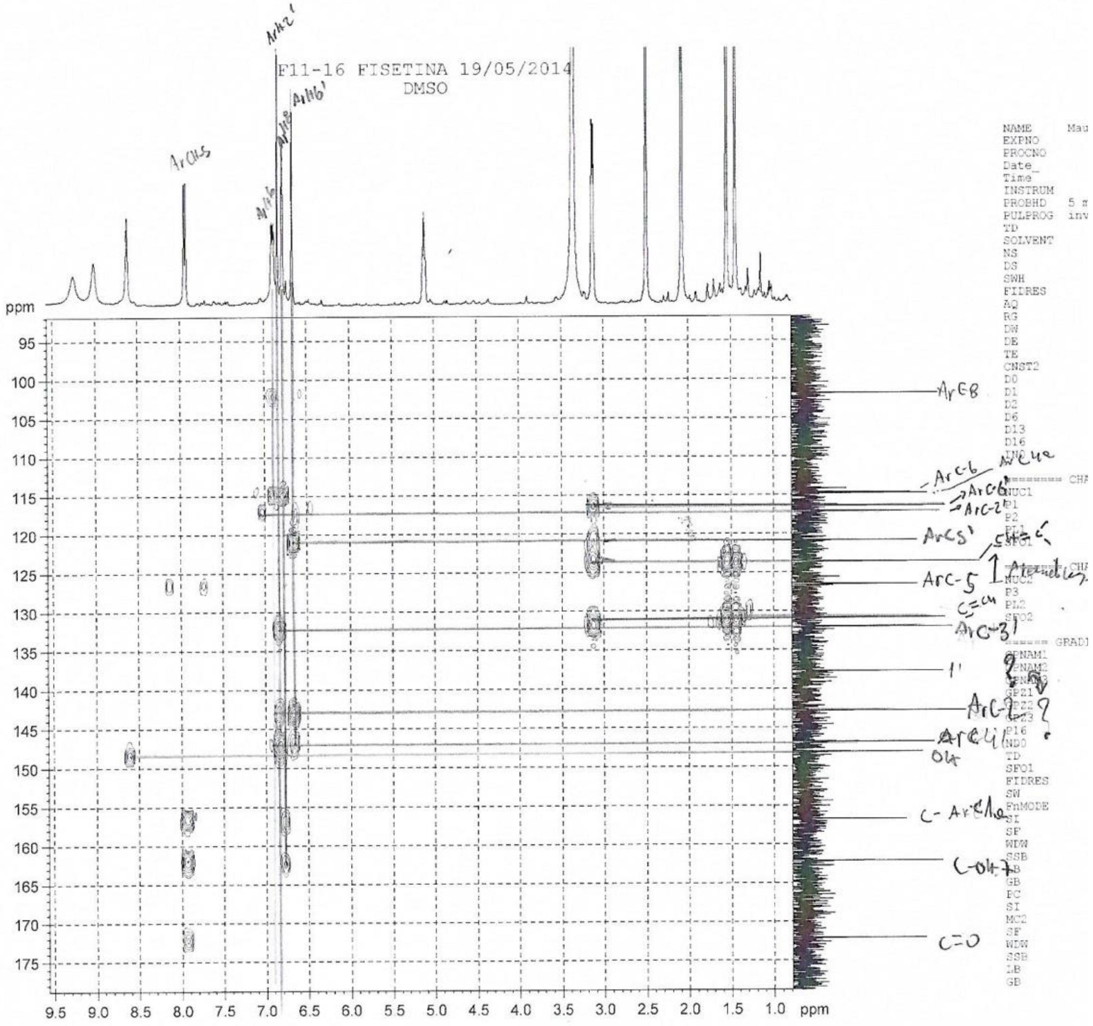

Figure S25. Zoom of 2D HMBC of **10**.

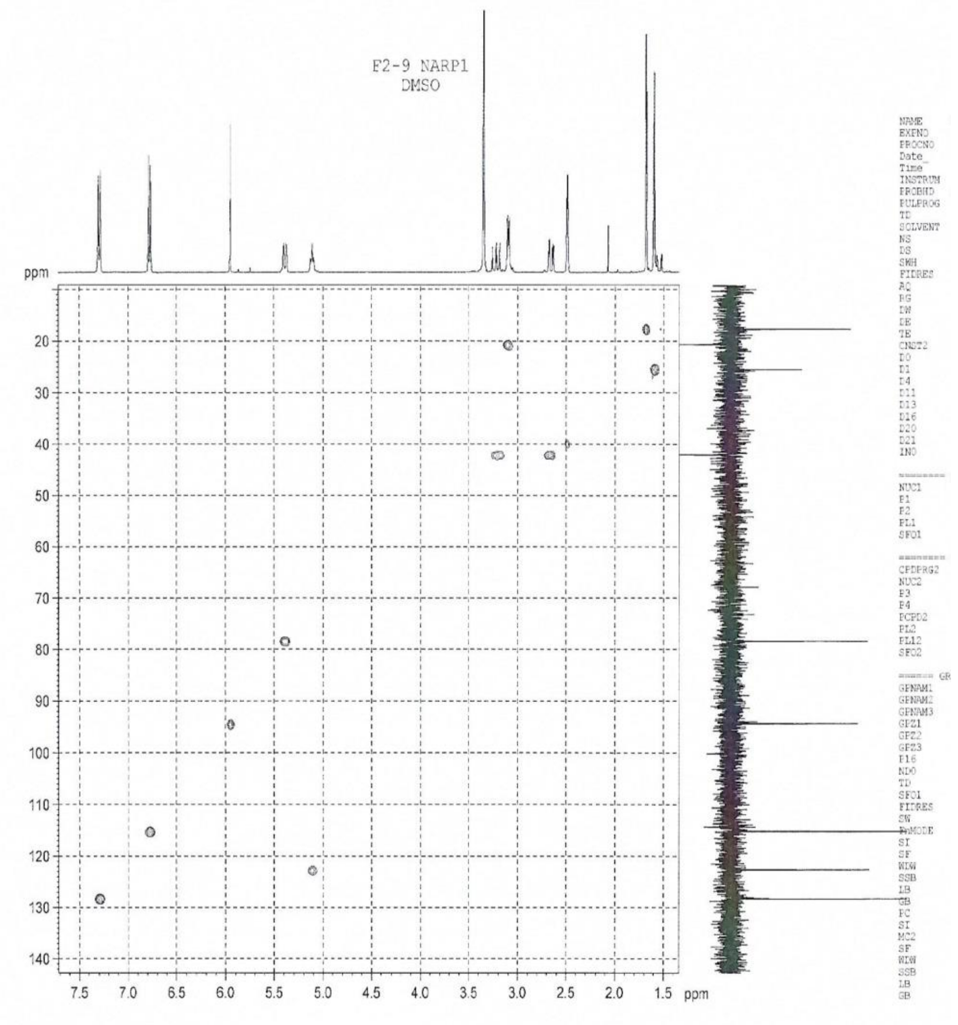

Figure S26. 2D HSQC of **11**.

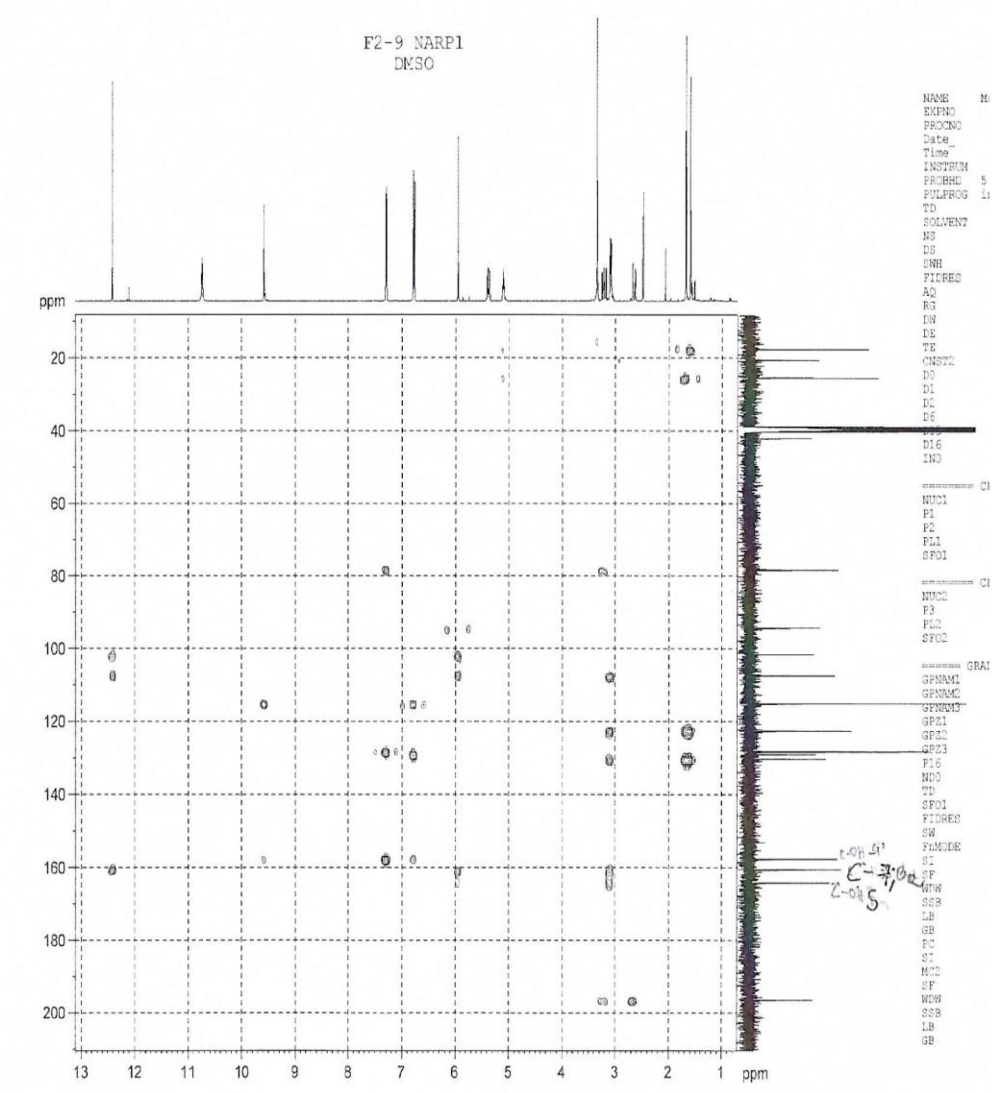

Figure S27. 2D HMBC of **11**.

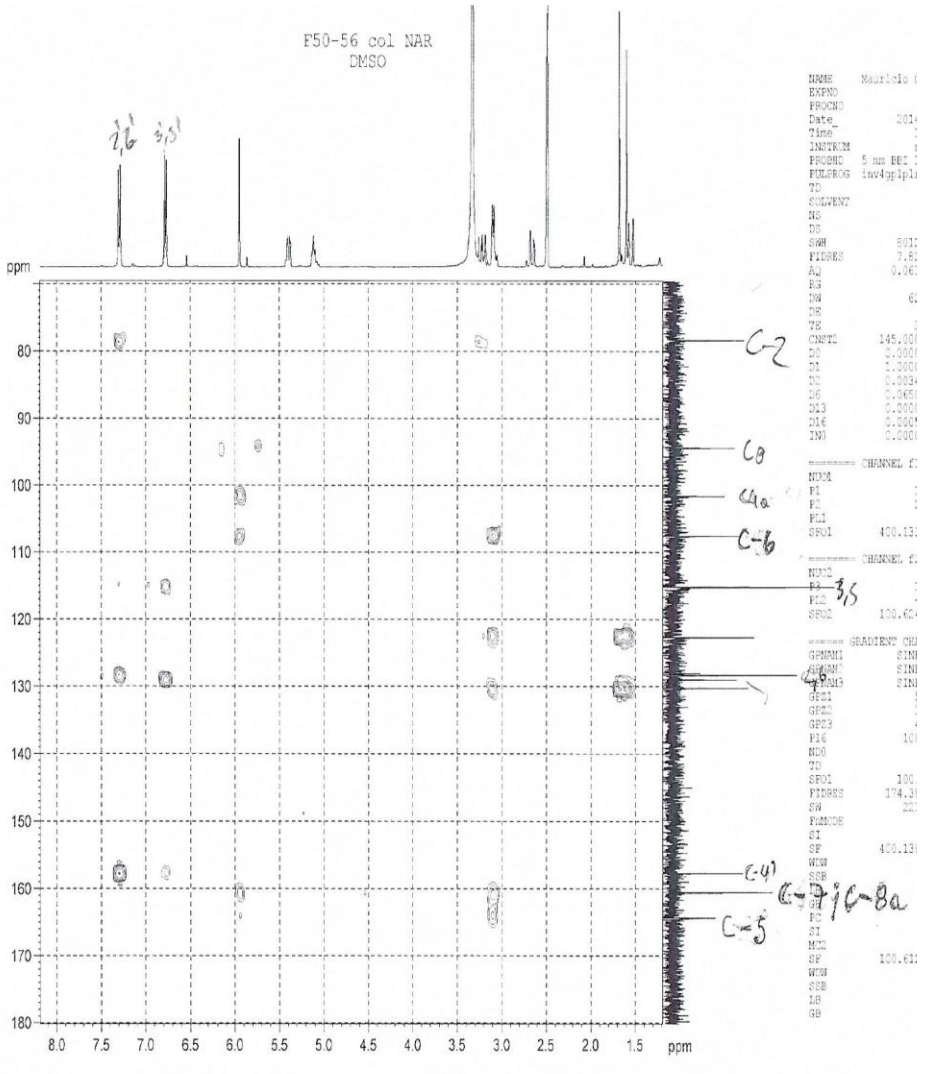

Figure S28. Zoom of 2D HMBC of **11**.

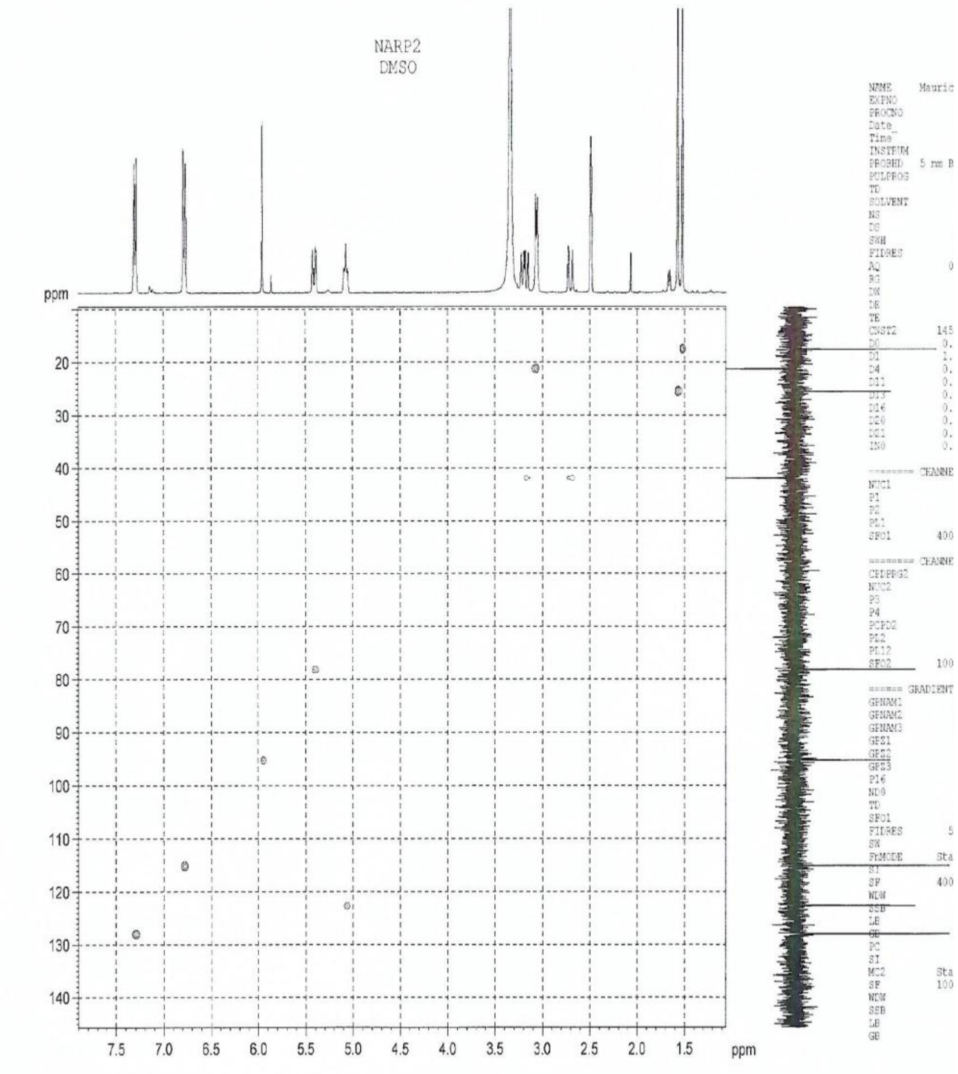

Figure S29. 2D HSQC of **12**.

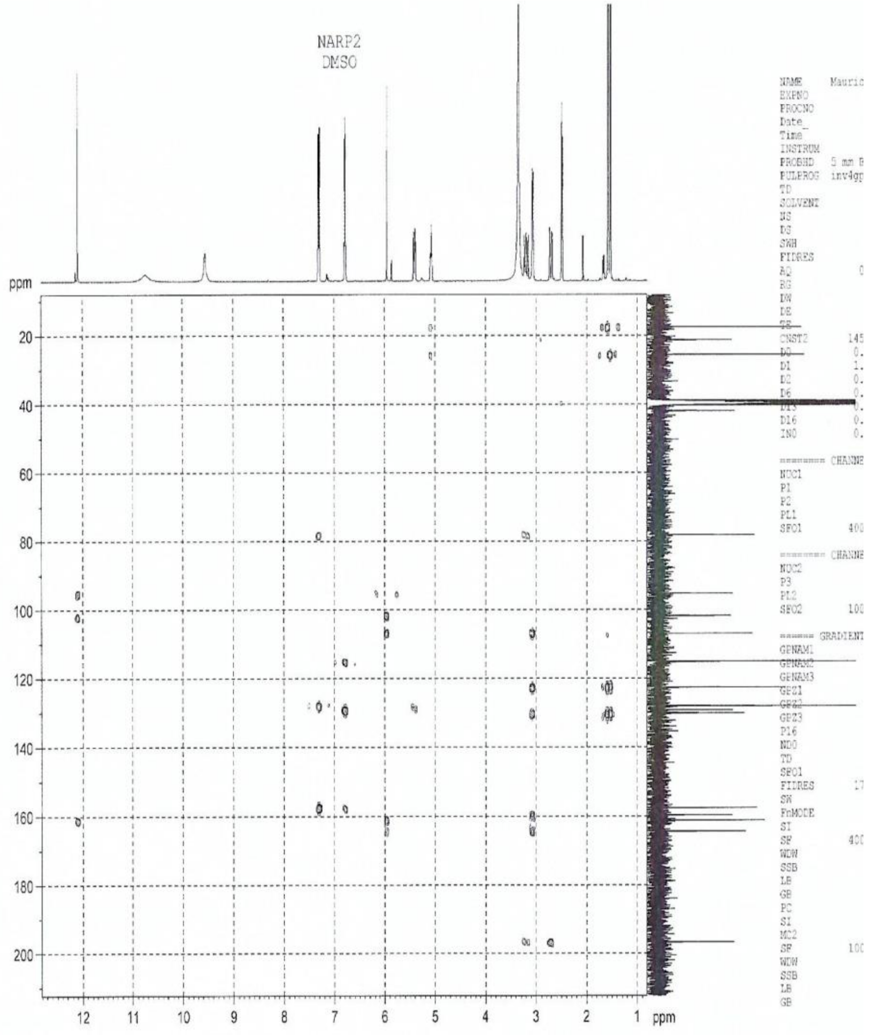

Figure S30. 2D HMBC of **12**.

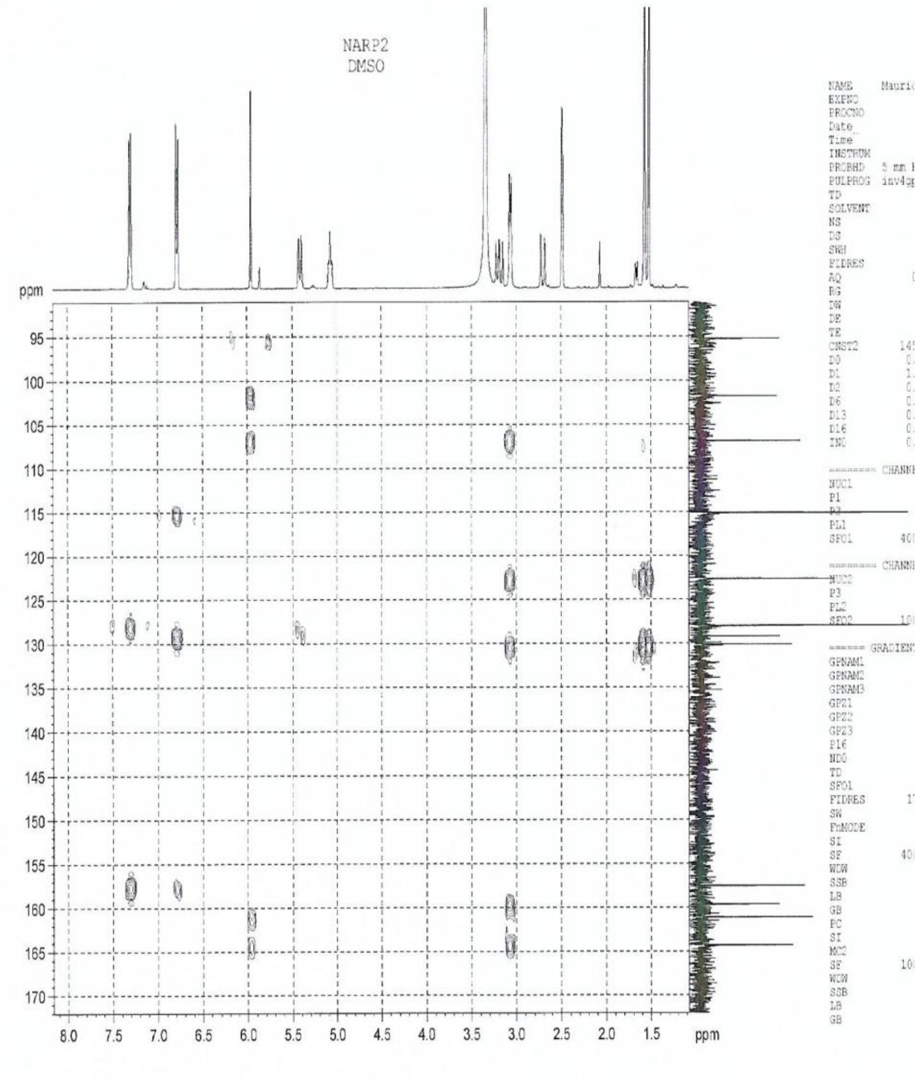

Figure S31. Zoom of 2D HMBC of **12**.

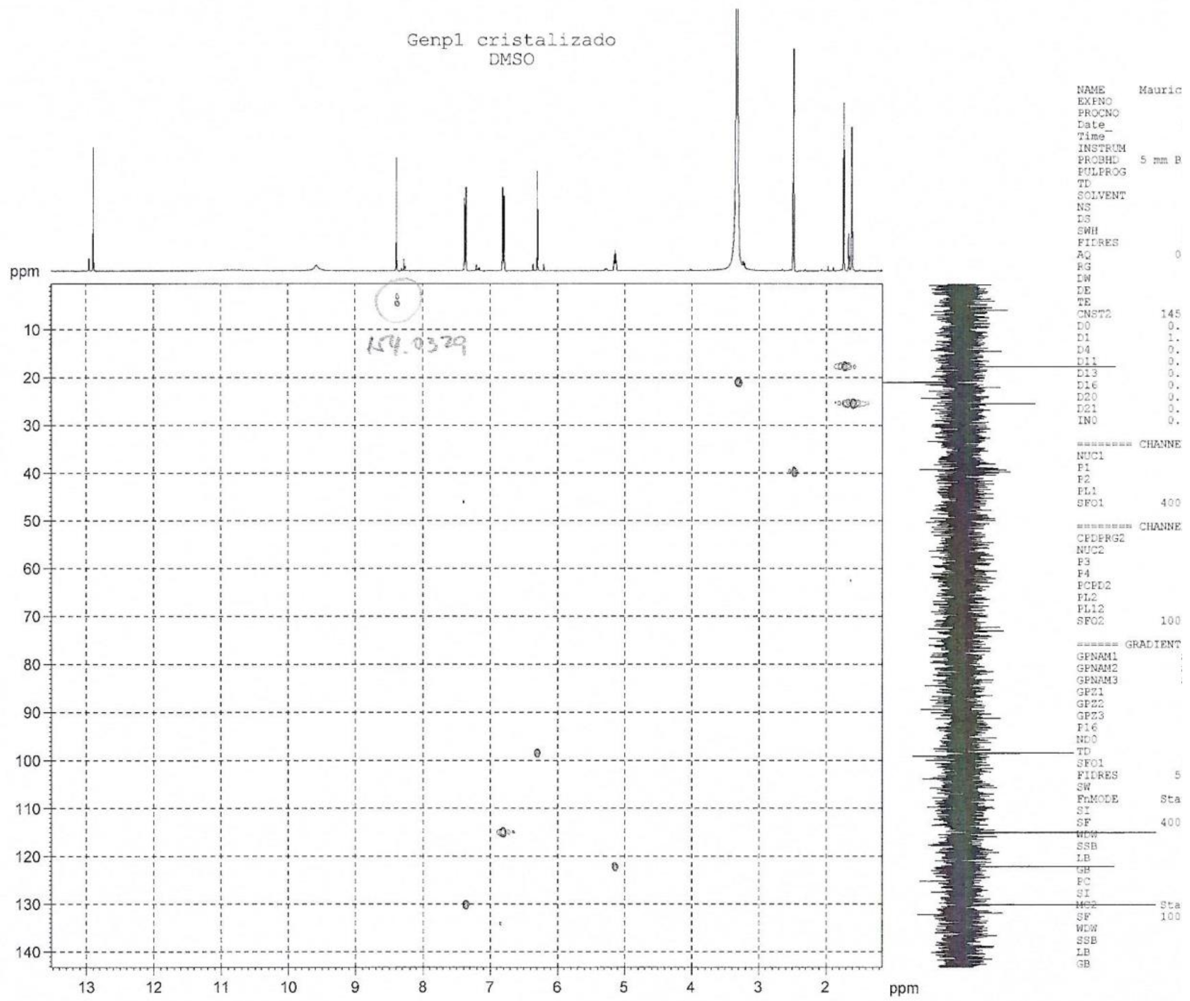

Figure S32. 2D HSQC of **13**.

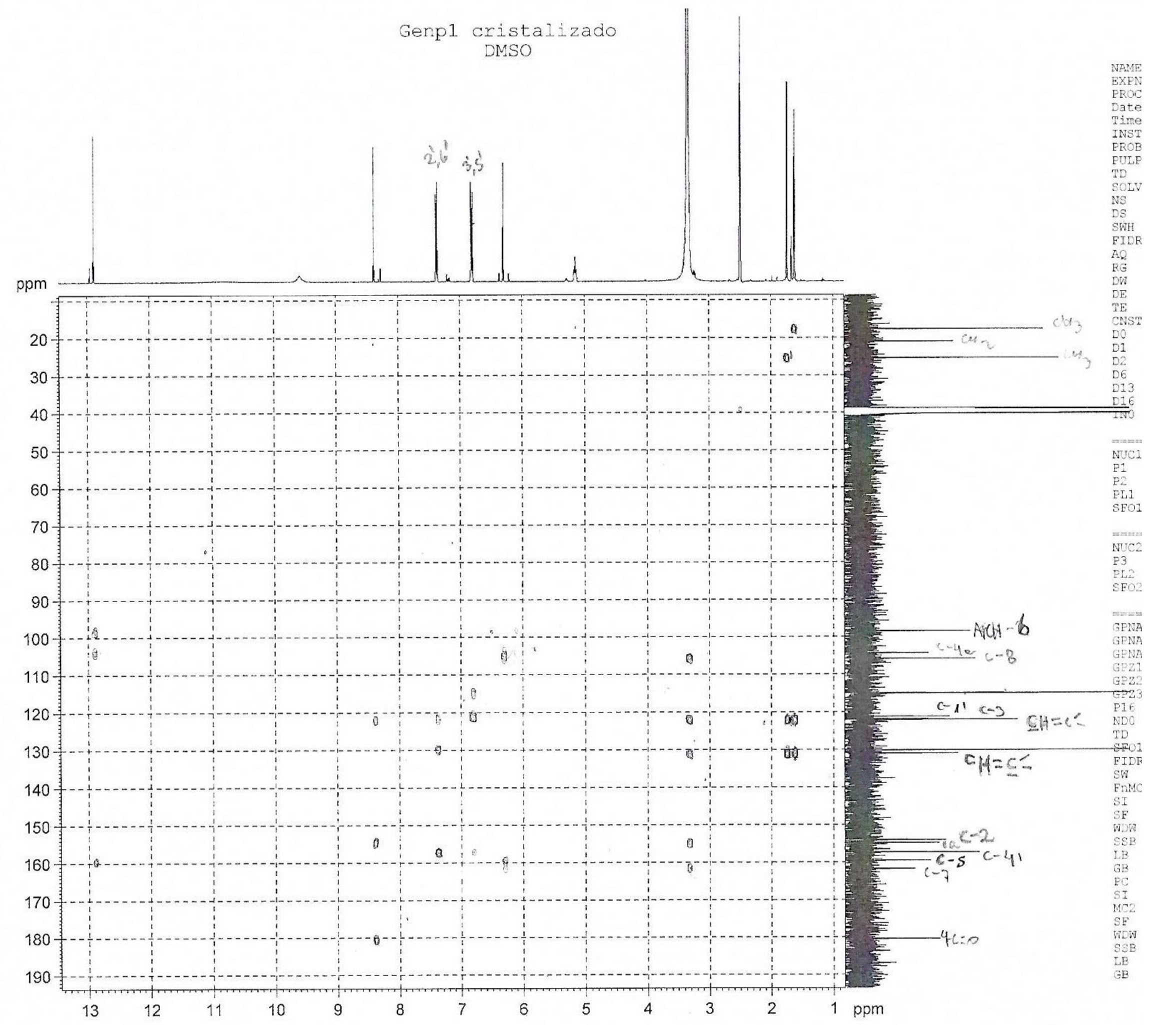

Figure S33. 2D HMBC of **13**.

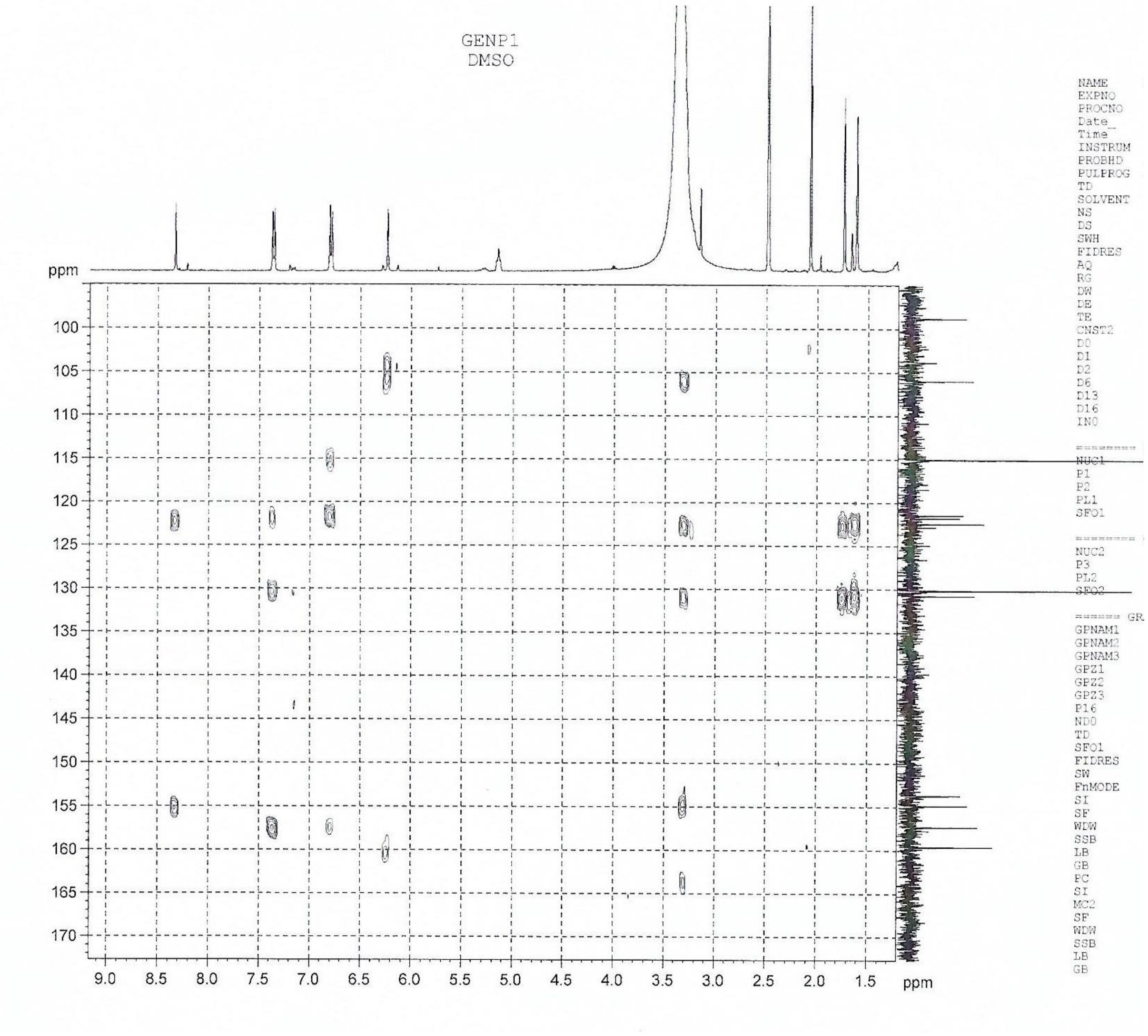

Figure S34. Zoom of 2D HMBC of **13**.
